# Supplementary material for: A Simulation-Based Approach to Severe Bronchospasm Complicated by Septic Shock
Source: MedEdPORTAL. 2026 Apr 7;22:11592. doi: 10.15766/mep_2374-8265.11592 (PMC13053521; doi:10.15766/mep_2374-8265.11592)
Supplement: Supplementary file 1 — Simulation Case with Critical Actions.docxSimulation Environmental Preparation List.docxPrebriefing Guide.docxData Slides.pptxDebriefing Guide.docxPostdebrief Handout.docxSimulation Evaluation Form.docx [file mep_2374-8265.11592-s001.zip › A. Simulation Case with Critical Actions.docx]

Appendix A. Simulation Case with Critical Actions

| **Appendix A: *MedEdPORTAL* Simulation Cas**  **SIMULATION CASE TITLE:** A Simulation-Based Approach to Severe Bronchospasm Complicated by Septic Shock  AUTHORS: Taylor Merritt, MD, Annesha Dutta, MD, Yarah Ghotmi, MD & Ngoc Van Horn, MD  **LEARNER AUDIENCE:** Pediatric Residents | |
| --- | --- |
| **PATIENT NAME:** Aaron Huffman  **PATIENT AGE:** 2 years old  **CHIEF COMPLAINT:** respiratory distress  **PHYSICAL SETTING:** Pediatric Emergency Department | |
|  | |
| **Brief Narrative Description of Case** | Aaron Huffman is a 2-year-old with a past medical history of eczema presents via EMS from an outside hospital to the children’s emergency department in respiratory distress. He develops worsening bronchospasm in the setting of pneumonia requiring breathing treatments, steroids, and antibiotics. This leads to near fatal bronchospasm and respiratory failure requiring BiPAP initiation. He develops concurrent uncompensated septic shock requiring fluid resuscitation and pressors. |
| **Primary Learning Objectives** | By the end of this activity, learners will report increased confidence in:   1. Identifying and treating a patient with near-fatal bronchospasm. 2. Creating an appropriate respiratory support plan for a patient with progression to respiratory failure. 3. Choosing appropriate resuscitation interventions for severe hypotension concerning for septic shock. |
| **Initial Prompt** | During prebrief: Aaron Huffman is a 2-year-old presenting via EMS from an outside hospital to the children’s emergency department with a pneumonia. Family is on the way.  From EMS Virtual Embedded Participant in Room: We have a 2-year-old male coming from an OSH. He was diagnosed with pneumonia and received Amoxicillin. He desatted to 85% at the outside ED and was placed on 2L NC. On our way here he started working harder to breathe. Family is en route currently. |

| Initial Presentation | | | |
| --- | --- | --- | --- |
| **Initial Vital Signs** | HR: 110, sinus  BP: 86/60 (MAP 69)  RR: 45  SpO2: 97% on 2L NC  T: 38.4C  Cap Refill: 2s | | |
| **Overall Setting and Appearance** | Learners are in the emergency department. They receive handoff from EMS who brought the patient from an outside hospital. The patient is on 2L NC. The mannequin was moderate increase WOB with abdominal breathing and subcostal retractions. He is crying and wheezing in all lung fields. GCS 15. OSH paperwork is placed on patient bed for learners to read. | | |
| **Standardized Participants (and Their Roles in the Room at Case Start)** | EMS Avatar (or SP/voice over from instructor): “We have a 2-year-old male coming from an OSH. He was diagnosed with PNA and received Amoxicillin. He desatted to 85% at the outside ED and was placed on 2L NC. On our way here he started working harder to breathe. Family is en route currently.” | | |
| **HPI** | No HPI available verbally. All information is given via EMS avatar or via OSH paperwork that is provided on patient bed. | | |
| **Past Medical/Surgical History** | **Medications** | **Allergies** | **Family History** |
| Eczema | No home medications | None | None |
| **Physical Examination** | | | |
| **General** | Crying. Laying in bed. | | |
| **HEENT** | PERRLA. Clear oropharynx. NC in place. | | |
| **Neck** | Supple. | | |
| **Lungs** | Expiratory wheezes in all lung fields. Moderate WOB with abdominal breathing and subcostal retractions. RR 45. | | |
| **Cardiovascular** | RRR, no murmur. | | |
| **Abdomen** | Soft, active bowel sounds. | | |
| **Neurological** | Crying, GCS 15. | | |
| **Skin** | No rashes. | | |

| Instructor Notes - Changes and CASE Branch Points |
| --- |

| Frame | Description | Vitals | Triggers & Critical Actions | Notes & Labs | Checklist |
| --- | --- | --- | --- | --- | --- |
| 1 | On 2L NC. Moderate increase WOB with abdominal breathing and subcostal retractions. Responding appropriately. Wheezing in all lung fields. GCS 15 (crying). | HR: 110, sinus  BP: 86/60 (MAP 69)  RR: 45  SpO2: 97% on 2L NC  T: 38.4C  Cap Refill: 2s | Trigger: Watch a video showing respiratory distress. Listen to wheezing.  Critical Action/If team:  Completes a physical exam, asks for PIV, and starts albuterol, ipratropium, steroids, and antibiotics → GO TO FRAME 2  If NO ACTION or no albuterol or no steroids, or no antibiotics → LIFESAVER → GO TO FRAME 2 | OSH Paperwork with printed CXR at bedside.  LIFESAVER: NURSE overhead: “He’s wheezing and febrile. Is there anything else that you want to do?”  VBG #1: 7.4/40/30 + lactate 1 | The Team provided:  [ ] albuterol  [ ] ipratropium  [ ] steroids  [ ] antibiotics |
| 2 | Patient becomes obtunded with increased WOB. GCS 10 and crying stops. | HR: 190  BP: 86/60 (MAP 69)  RR: 65  SpO2: 84%  T: 37.8C  Cap Refill: 2s | Trigger: Change in mental status as patient stops crying  Critical Action/If team:  If continuous albuterol, steroids, BiPAP, Mag with bolus → GO TO FRAME 3  If continuous albuterol, steroids, BiPAP, Mag w/o bolus → GO TO FRAME 3  If continuous albuterol + steroids + BiPAP, but NO MAG → GO TO FRAME 3  If NO action or no BiPAP, LIFESAVER → GO TO FRAME 3 | LIFESAVER: NURSE overhead: “He’s breathing hard, anything else?”  VBG #2: 7.1/60/30 + lactate 2  POC Glucose: 135  If team asks for repeat CXR, overhead to say XR is delayed.  If team asks for intubation supplies, overhead to say that we don’t have supplies currently. | The Team provided:  [ ] continuous albuterol  [ ] BiPAP  [ ] Magnesium  [ ] Bolus  The Team verbalized:  [ ] diagnosis of bronchospasm  [ ] BiPAP settings |
| 3 | Patient's WOB & mentation improve (crying). Wheezing resolves. GCS 15. | HR: 198  BP: 68/31 (MAP 43)  RR: 35  SpO2: 92%  Cap Refill: 5s  T: 37.8C | Trigger: hypotension with widened pulse pressure.  Critical Action/If team:  If 2nd fluid bolus + vasopressor → BP returns to 86/60 (MAP 69) → TRANSFER TO PICU → END OF SIM  If NO ACTION or fluid bolus only or pressor bolus only → LIFESAVER → TRANSFER TO PICU → END OF SIM | LIFESAVER: VS changing with worsening hypotension with BP 60/25 (MAP 37).  VBG #3: 7.3/44/20 + lactate 4  PICU to answer call and accept patient after handoff. | The team provided:  [ ] 2^nd^ bolus  [ ] vasopressor  The Team verbalized:  [ ] diagnosis of septic shock |

**Ideal Scenario Flow**

The learners enter the room to find a 2-year-old patient in moderate respiratory distress with wheezing on auscultation. They receive handoff from EMS. They place the patient on bedside monitors and obtain an IV. The patient is given albuterol, ipratropium, corticosteroids, and antibiotics. Despite initial interventions, the patient’s respiratory distress worsens, with increased work of breathing and wheezing. Learners escalate to continuous albuterol, magnesium with a fluid bolus, and BiPAP in response to rising CO₂ levels and worsening mental status. During this time, the patient becomes increasingly hypotensive though their respiratory status improves. A second fluid bolus is administered, but the hypotension and poor perfusion persist. The team initiates broad-spectrum antibiotics and recognizes the need for vasopressor support, selecting norepinephrine or vasopressin for suspected septic shock. The patient’s vital signs stabilize following initiation of vasopressors and continued respiratory support. The team communicates with the PICU and prepares the patient for admission.

**Anticipated Management Mistakes**

1. Failure to escalate respiratory support in a timely manner: Some learners continued to provide nebulized treatments and supplemental oxygen despite signs of worsening respiratory failure (e.g., rising CO₂, increased work of breathing). We found that allowing the patient’s condition to deteriorate until BiPAP is initiated helped reinforce the need for early escalation in near-fatal bronchospasm.
2. Delayed recognition of septic shock: Learners often focused on the asthma component and failed to recognize evolving hypotension and poor perfusion as signs of septic shock. We adjusted the scenario timing to stagger respiratory and hemodynamic decline, prompting learners to address both processes independently.
3. Uncertainty in selecting the appropriate pressor agent: Learners were often unsure which vasopressor to initiate based on the patient’s clinical presentation. To address this, we incorporated targeted debriefing on pediatric shock types and vasoactive agent selection, supported by a visual reference in subsequent sessions.
4. Difficulty applying and operating respiratory support equipment: Several learners were unfamiliar with the setup and proper application of respiratory interfaces. To address this, we incorporated this into the debrief as needed.


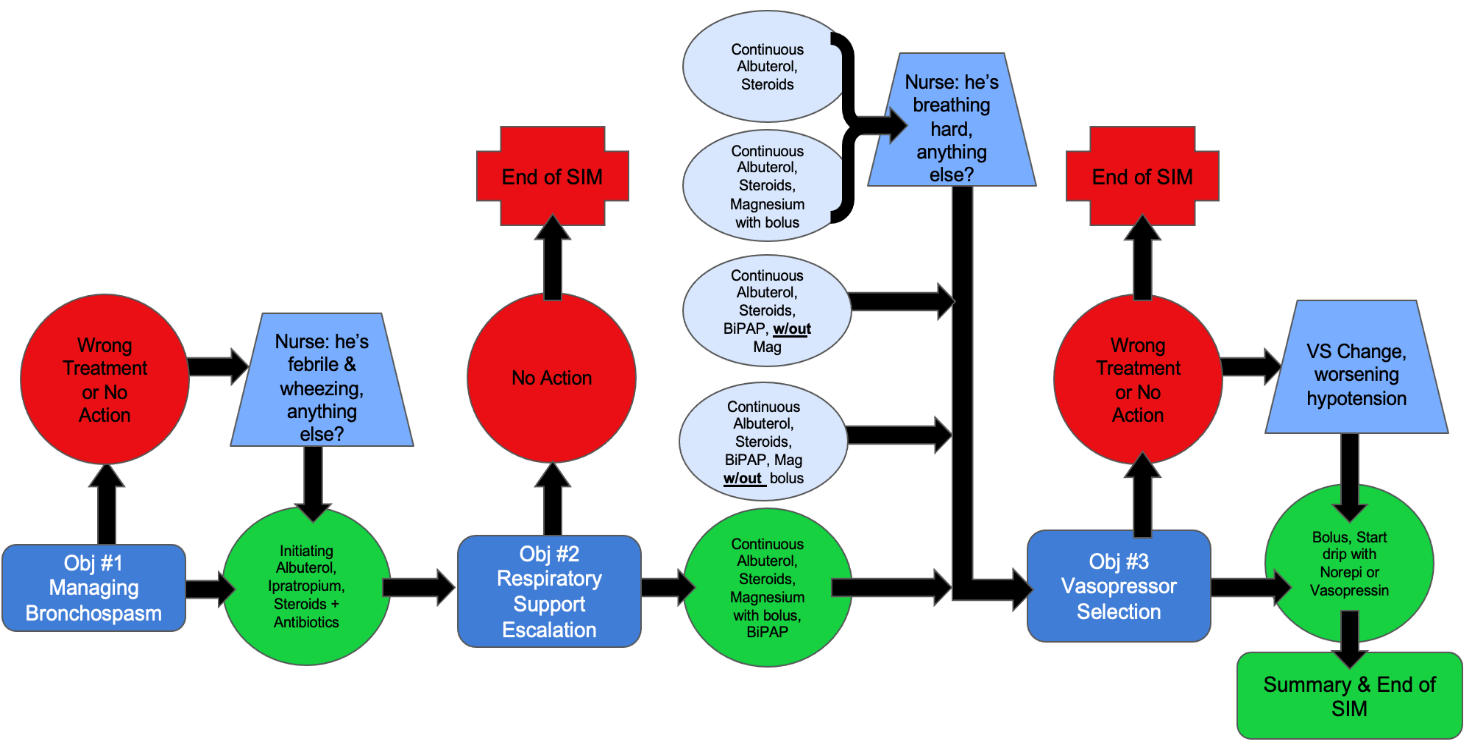


Image 1: Critical Action Flowsheet. Author owned.
