# Supplementary material for: A Simulation-Based Approach to Severe Bronchospasm Complicated by Septic Shock
Source: MedEdPORTAL. 2026 Apr 7;22:11592. doi: 10.15766/mep_2374-8265.11592 (PMC13053521; doi:10.15766/mep_2374-8265.11592)
Supplement: Supplementary file 1 — Simulation Case with Critical Actions.docxSimulation Environmental Preparation List.docxPrebriefing Guide.docxData Slides.pptxDebriefing Guide.docxPostdebrief Handout.docxSimulation Evaluation Form.docx [file mep_2374-8265.11592-s001.zip › B. Simulation Environmental Preparation List.docx]

**Appendix B.** **Simulation Environmental Preparation List**

**Resources**

- PALS reference cards
- Broselow Tape

**Simulated Medications** (bolded medications are more likely to be used in this simulation)

- **Acetaminophen**
- **Ceftriaxone**
- Epinephrine
- Ketamine
- Lactated Ringer’s
- **Norepinephrine**
- **Normal Saline (NS)**
- Ondansetron
- Ibuprofen
- **Vancomycin**
- **Magnesium Sulfate**
- Solumedrol
- Prednisolone
- **Dexamethasone**
- Terbutaline
- Aminophylline
- **Albuterol**
- **Ipratropium**
- Levalbuterol
- Vasopressin
- Dobutamine
- Dopamine
- Milrinone

**Equipment**

- High Fidelity mannikin in hospital gown on bed that can display evolving physical exam findings, including wheezing and poor perfusion.
- Monitor – Noninvasive blood pressure (NIBP), Heart Rate (HR), Respiratory Rate (RR), Oxygen saturation (SpO2), temperature (T)
- Blood Pressure cuff, Heart Rate monitor leads, oxygen saturation probe
- Oxygen hook-up on wall or cylinder
- Bag-valve-mask system
- Nasal cannula (simple and high flow)
- Non-rebreather mask
- BiPAP mask
- Suction device
- Shoulder roll
- Stethoscopes
- IV tubing and filters, IV pumps, pressure bags

**Additional specific equipment for this scenario:**

- Video screen access for Avatar, acute respiratory distress video, and lab results
- Outside Hospital discharge paperwork w/ CXR
