# Supplementary material for: A Simulation-Based Approach to Severe Bronchospasm Complicated by Septic Shock
Source: MedEdPORTAL. 2026 Apr 7;22:11592. doi: 10.15766/mep_2374-8265.11592 (PMC13053521; doi:10.15766/mep_2374-8265.11592)
Supplement: Supplementary file 1 — Simulation Case with Critical Actions.docxSimulation Environmental Preparation List.docxPrebriefing Guide.docxData Slides.pptxDebriefing Guide.docxPostdebrief Handout.docxSimulation Evaluation Form.docx [file mep_2374-8265.11592-s001.zip › C. Prebriefing Guide.docx]

**Appendix C. Prebriefing Guide**

**SIM Pre-Briefing Script**

**Introduction (2 minutes):** Today we will be participating in a Simulation. I would like to start with some introductions. Would you mind going around the room and saying your name, year, and career interest? My role today is to facilitate this experience and promote reflection on your actions and decision-making in this scenario.

**Goals of the SIM are to be ready to:**

- Correctly triage and manage a child presenting from an OSH with minimal handoff.
- Verbalize a differential diagnosis and management plan.
- Work in a collaborative fashion with our pharmacy colleagues and escalate patient management as needed.

**Debriefing:** We will be starting with a 10-minute pre-brief, the simulation for 15 minutes, and then a 25 min debriefing session. During the debriefing, I will ask more about your decision-making process and the thoughts that drove your actions and decisions. I may also ask about teamwork, communication, leadership, and team roles as you adapted to the situation.

**Assessment:** Our purpose here today is for learning and development. There will not be any grading or reporting about your performance today. This activity gives you a chance to practice and learn in a safe environment.

**Basic Assumption:** I believe everyone here is a capable and intelligent human who wants to improve. I believe that mistakes are a part of learning and will not be punished or judged. We are all training and continually learning. Mistakes are welcome and help us improve.

**Fiction Contract:** We know that there are many gaps between this simulation and the reality of patient care. Please try your best to suspend realism and immerse yourself in the situation to make the most of this learning opportunity. **In this specific scenario, there will be media images to try to best recreate physical exam findings. If you need clarification, please ask the facilitator.** We know they are not perfect, but please try your best to “buy in” to the overall clinical presentation.

**Confidentiality:** We ask that you are mindful of confidentiality during this simulation day. What happens in sim stays in sim. Please don’t discuss your colleague’s performance outside of the case and please do not discuss details of this case with fellow residents. We re-use these cases and would like everyone to have the same experience without any kind of advantages going into the scenario.

**Safety:** There are some additional safety considerations I want to review. In the unlikely event that something “real” happens, such as a fire alarm or a clinical emergency, one of the faculty/staff will say: “This is not a simulation.” If you hear those words that means that this is a real event and that you should respond accordingly. Additionally, in this scenario there is nothing you can do that will “kill” the patient.

**Roles:** Please organize your team into roles and assign responsibilities as you see fit. There will need to be one team leader. Your scope of practice will reflect the same scope of practice expected from a resident physician at your level of training. There will be no one assigned to a role that they are not trained to be (for example, a resident will not be assigned as an RT, instead they will be a doctor on airway).

Any questions before we move on to our simulation today?

**Simulation Considerations:** The simulation will take place entirely in the Emergency Department. In this scenario, you are at an academic pediatric hospital with in-house PICU, consults, anesthesia, and surgery teams. You may verbalize making consults, calling a code, etc. to utilize these resources or for extra guidance during this scene.

**Virtual Embedded Simulation Performer (ESP)**: There will be a virtual ESP via Avatar: EMS. The avatar will guide you as needed throughout the scenario, please take their concerns or questions seriously.

**Orientation to the Case**

- The crash cart and all necessary Items will be present, the drawers will be taped for any materials that you will not need. All equipment will be near the bedside or in the crash cart (nothing in the cabinets).
- Pharmacy Table will be present. All medications will be in the room and drawn in real time.
- The OSH discharge summary will be available to you on the pharmacy table when you enter.
- TV screen/Prompt: If you request labs or imaging, they will appear on the screen as relevant. Please request each lab individually. **Please verbally interpret all labs and images within the discharge summary and on the TV screen, anyone can provide this interpretation.** Changing physical exam findings will also appear on the screen, you may consider designating one person to continually check the TV screen for changes.
- The manikin will have pulses, breath sounds, and microphone capability. **Please be aware that this will continually change through the scenario.** The control room will notify you if there are any technical difficulties with the manikin or monitors.
- **In this scenario, there will be frequent changes in the patient’s physical exam. If you are unsure of what the changes are, please ask for clarification. As part of our fiction contract as stated earlier, this is simulation and we will be using simulation time here so please try your best to “buy in” to the timing as much as possible.**
- Please do not remove any drapes unless instructed by the facilitator.

Any questions (1-2 minutes)?

**Prompt**: Aaron Huffman is a 2-year-old presenting via EMS from OSH to the children’s ED in PNA. Family is on the way.
