# Supplementary material for: A Simulation-Based Approach to Severe Bronchospasm Complicated by Septic Shock
Source: MedEdPORTAL. 2026 Apr 7;22:11592. doi: 10.15766/mep_2374-8265.11592 (PMC13053521; doi:10.15766/mep_2374-8265.11592)
Supplement: Supplementary file 1 — Simulation Case with Critical Actions.docxSimulation Environmental Preparation List.docxPrebriefing Guide.docxData Slides.pptxDebriefing Guide.docxPostdebrief Handout.docxSimulation Evaluation Form.docx [file mep_2374-8265.11592-s001.zip › D. Data Slides.pptx]

## Slide 1
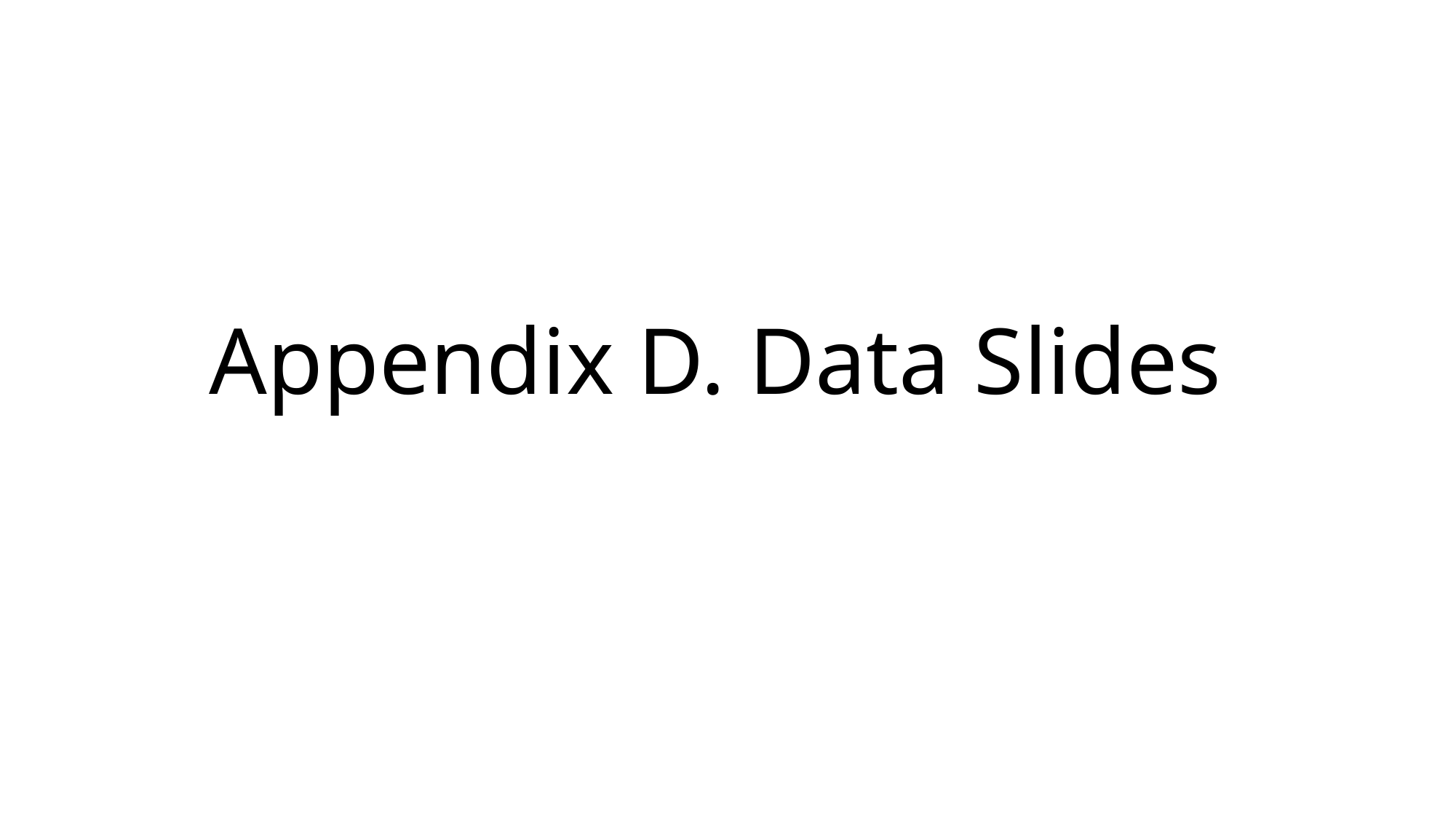

# Appendix D. Data Slides

## Slide 2
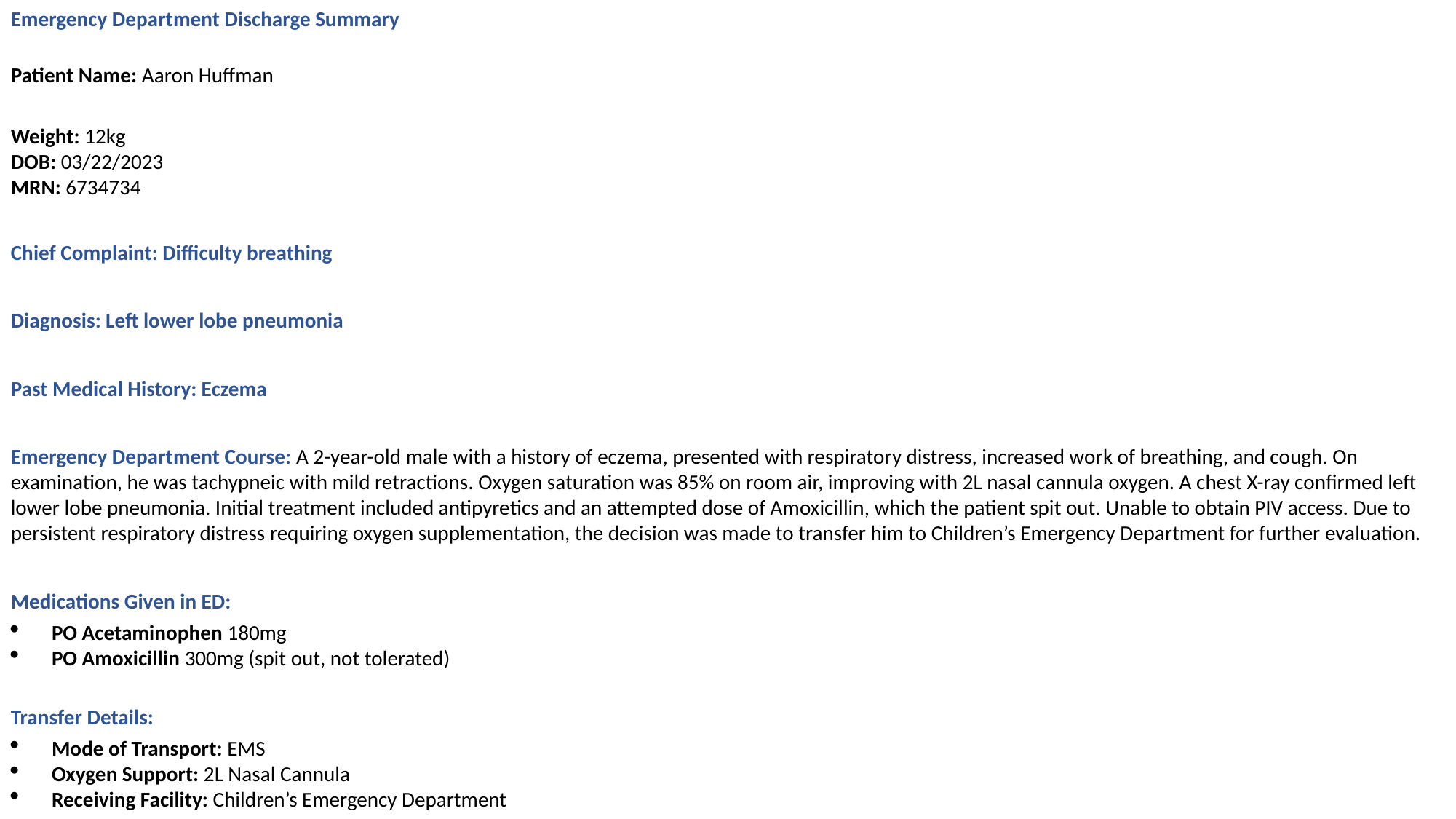

Emergency Department Discharge Summary
Patient Name: Aaron Huffman
Weight: 12kg
DOB: 03/22/2023
MRN: 6734734
Chief Complaint: Difficulty breathing
Diagnosis: Left lower lobe pneumonia
Past Medical History: Eczema
Emergency Department Course: A 2-year-old male with a history of eczema, presented with respiratory distress, increased work of breathing, and cough. On examination, he was tachypneic with mild retractions. Oxygen saturation was 85% on room air, improving with 2L nasal cannula oxygen. A chest X-ray confirmed left lower lobe pneumonia. Initial treatment included antipyretics and an attempted dose of Amoxicillin, which the patient spit out. Unable to obtain PIV access. Due to persistent respiratory distress requiring oxygen supplementation, the decision was made to transfer him to Children’s Emergency Department for further evaluation.
Medications Given in ED:
PO Acetaminophen 180mg
PO Amoxicillin 300mg (spit out, not tolerated)
Transfer Details:
Mode of Transport: EMS
Oxygen Support: 2L Nasal Cannula
Receiving Facility: Children’s Emergency Department

## Slide 3
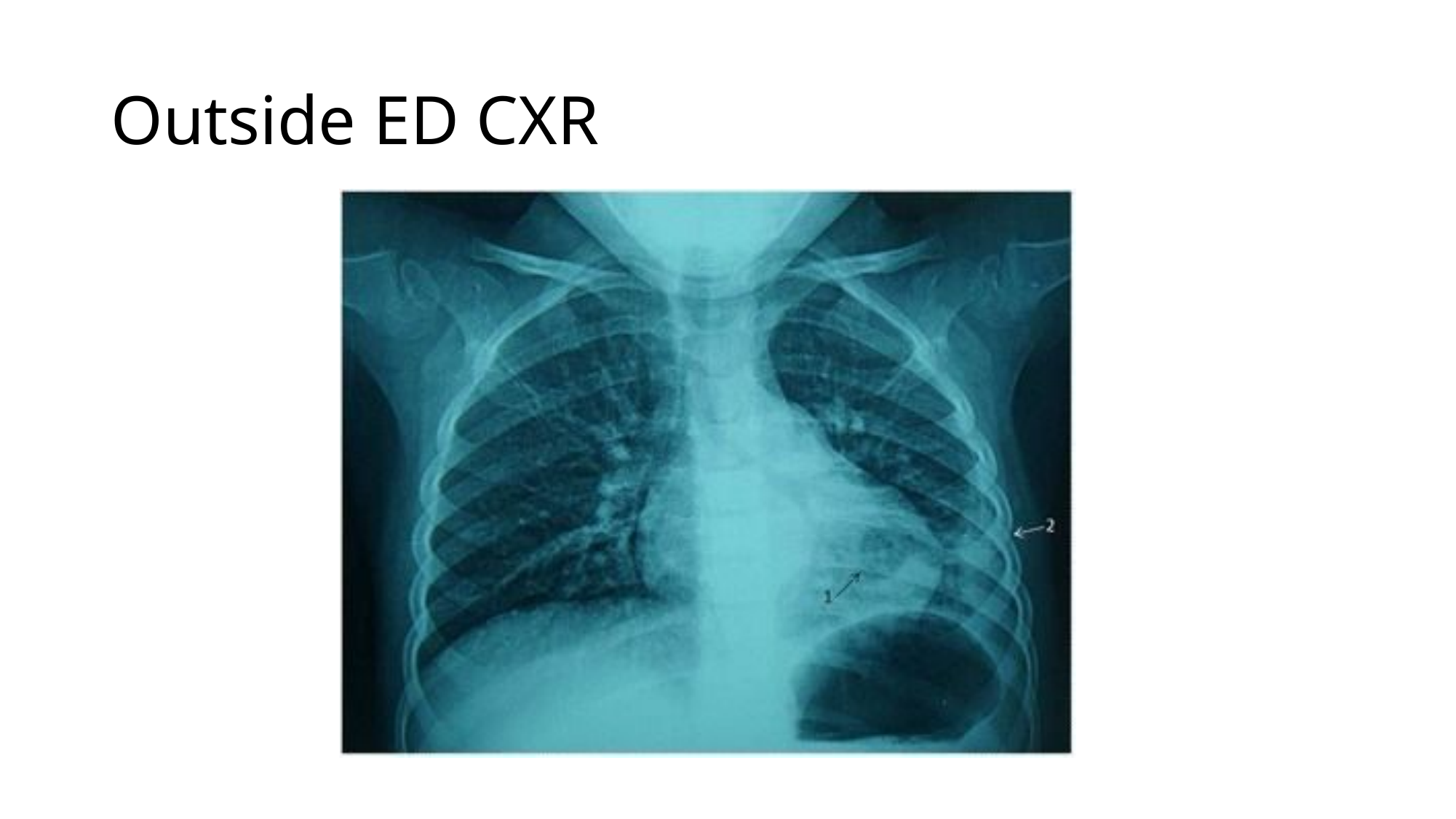

# Outside ED CXR

## Slide 4
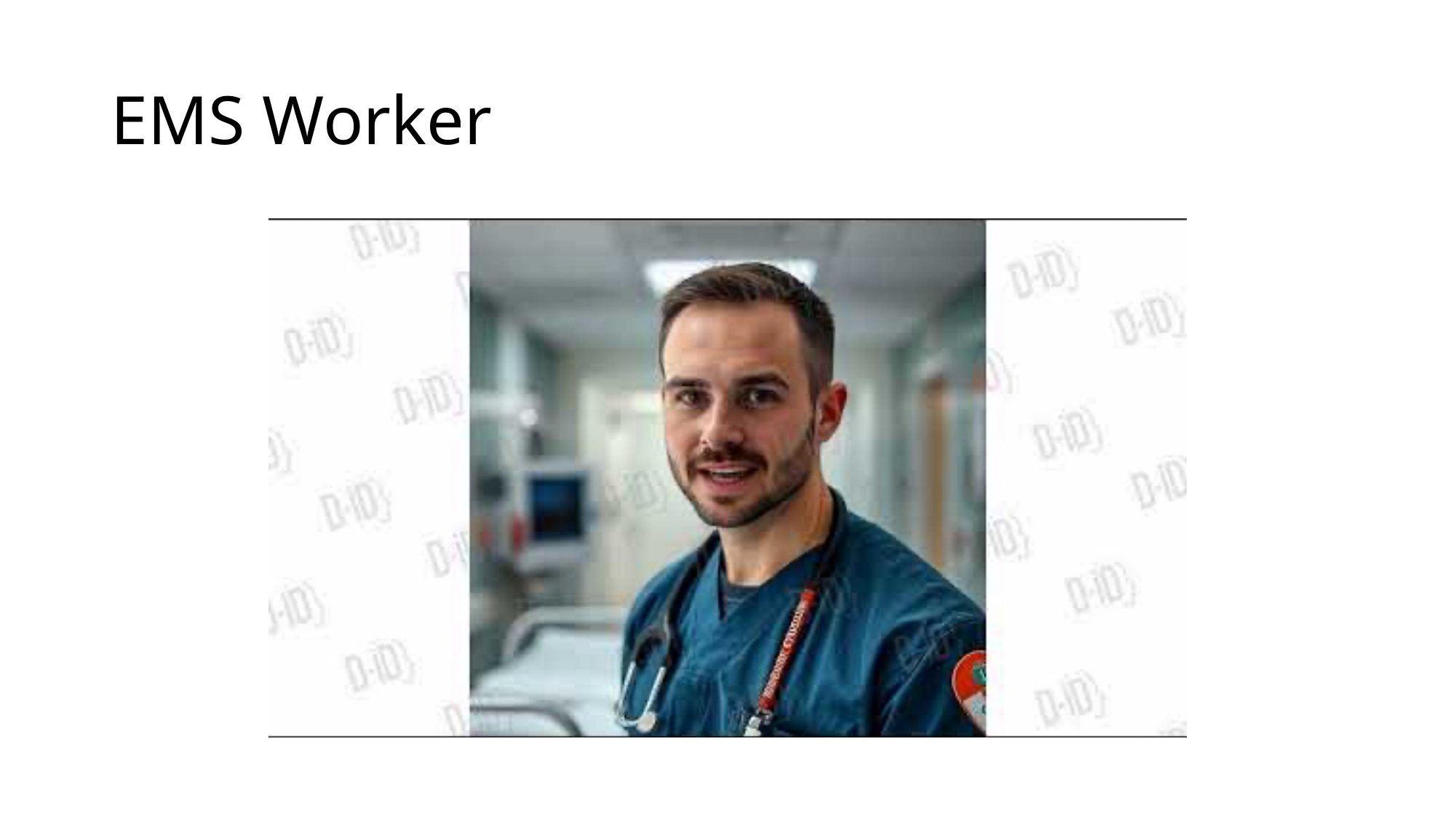

# EMS Worker

## Slide 5
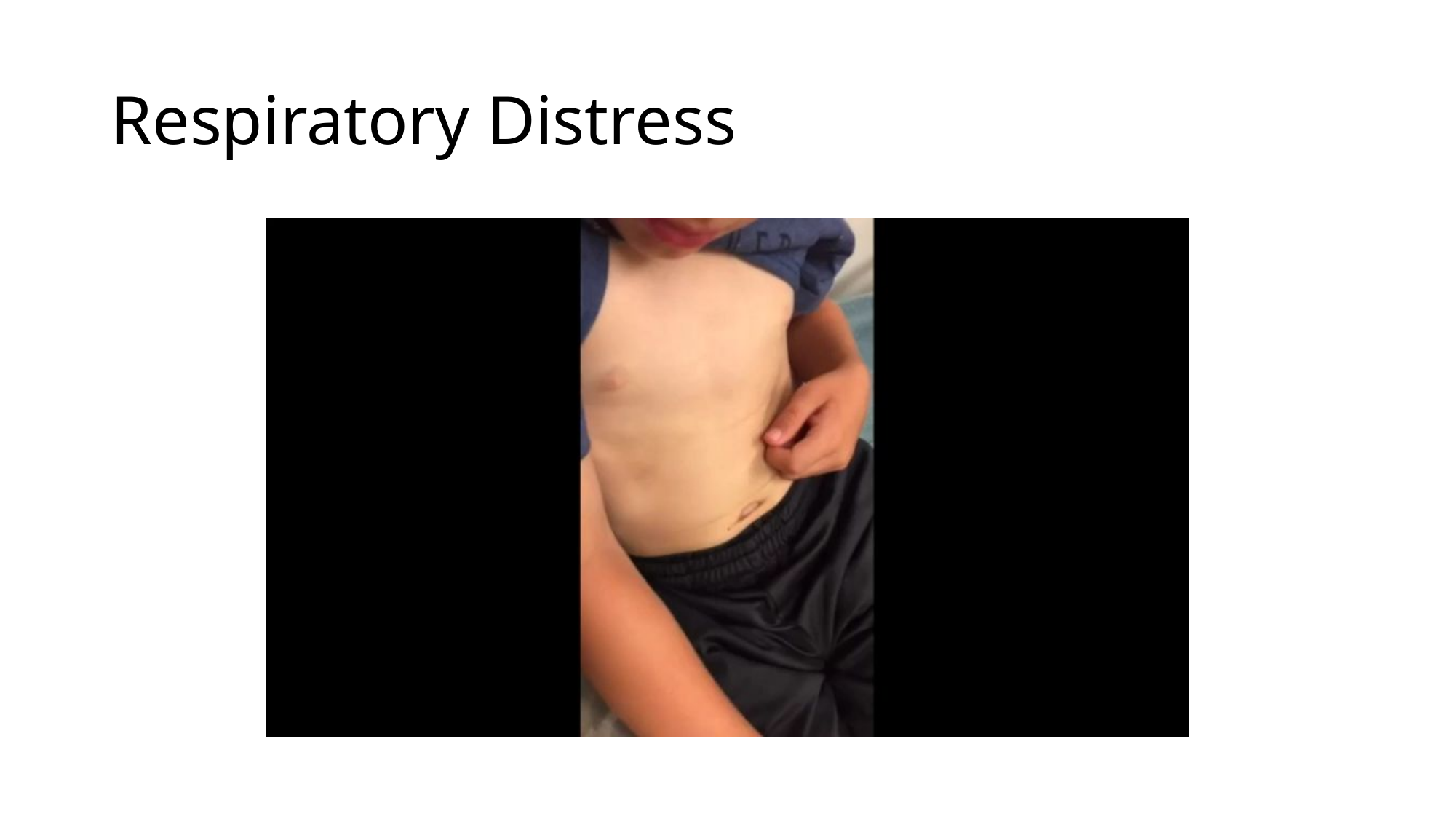

# Respiratory Distress

## Slide 6
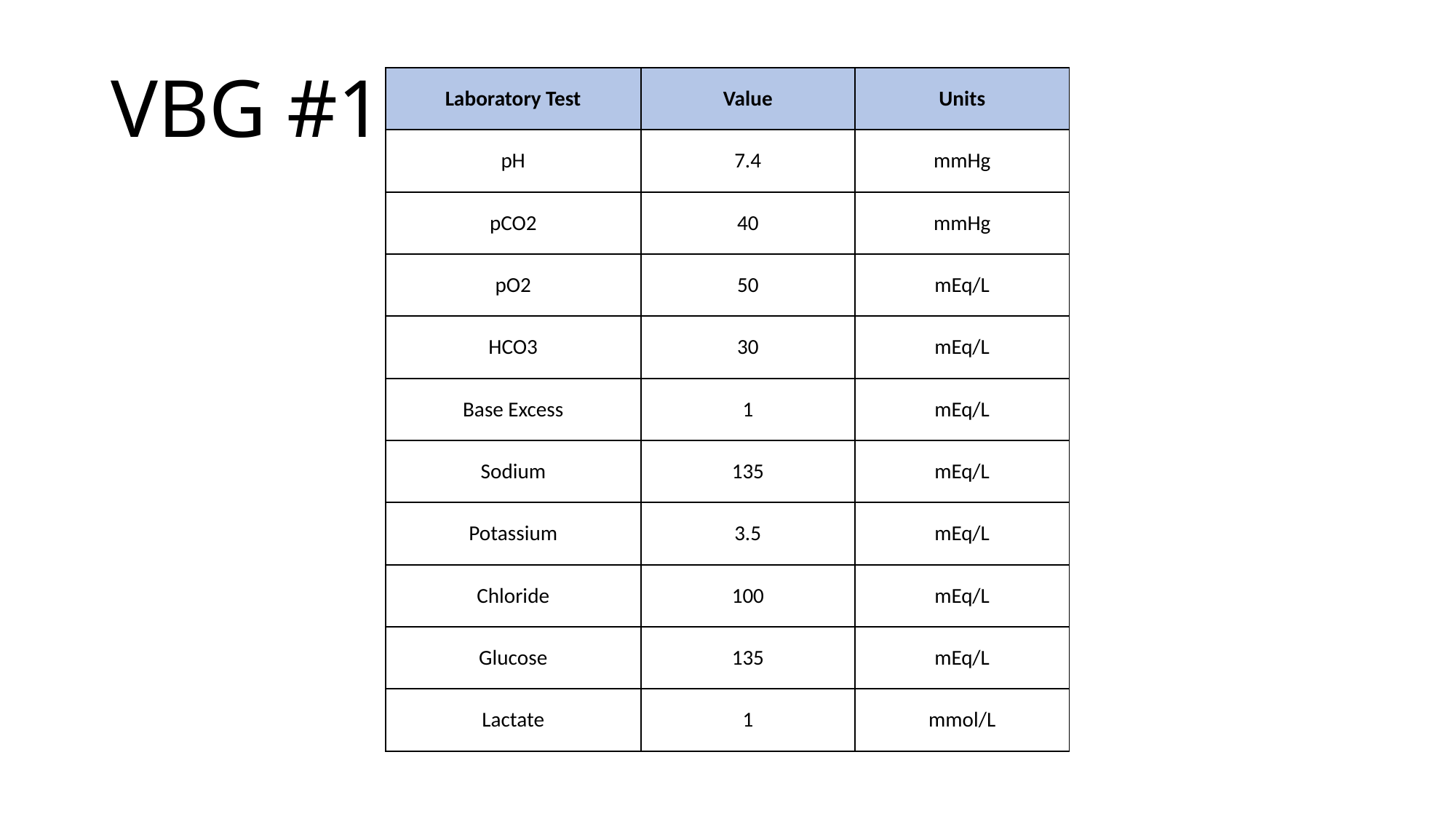

# VBG #1
| Laboratory Test | Value | Units |
| --- | --- | --- |
| pH | 7.4 | mmHg |
| pCO2 | 40 | mmHg |
| pO2 | 50 | mEq/L |
| HCO3 | 30 | mEq/L |
| Base Excess | 1 | mEq/L |
| Sodium | 135 | mEq/L |
| Potassium | 3.5 | mEq/L |
| Chloride | 100 | mEq/L |
| Glucose | 135 | mEq/L |
| Lactate | 1 | mmol/L |

## Slide 7
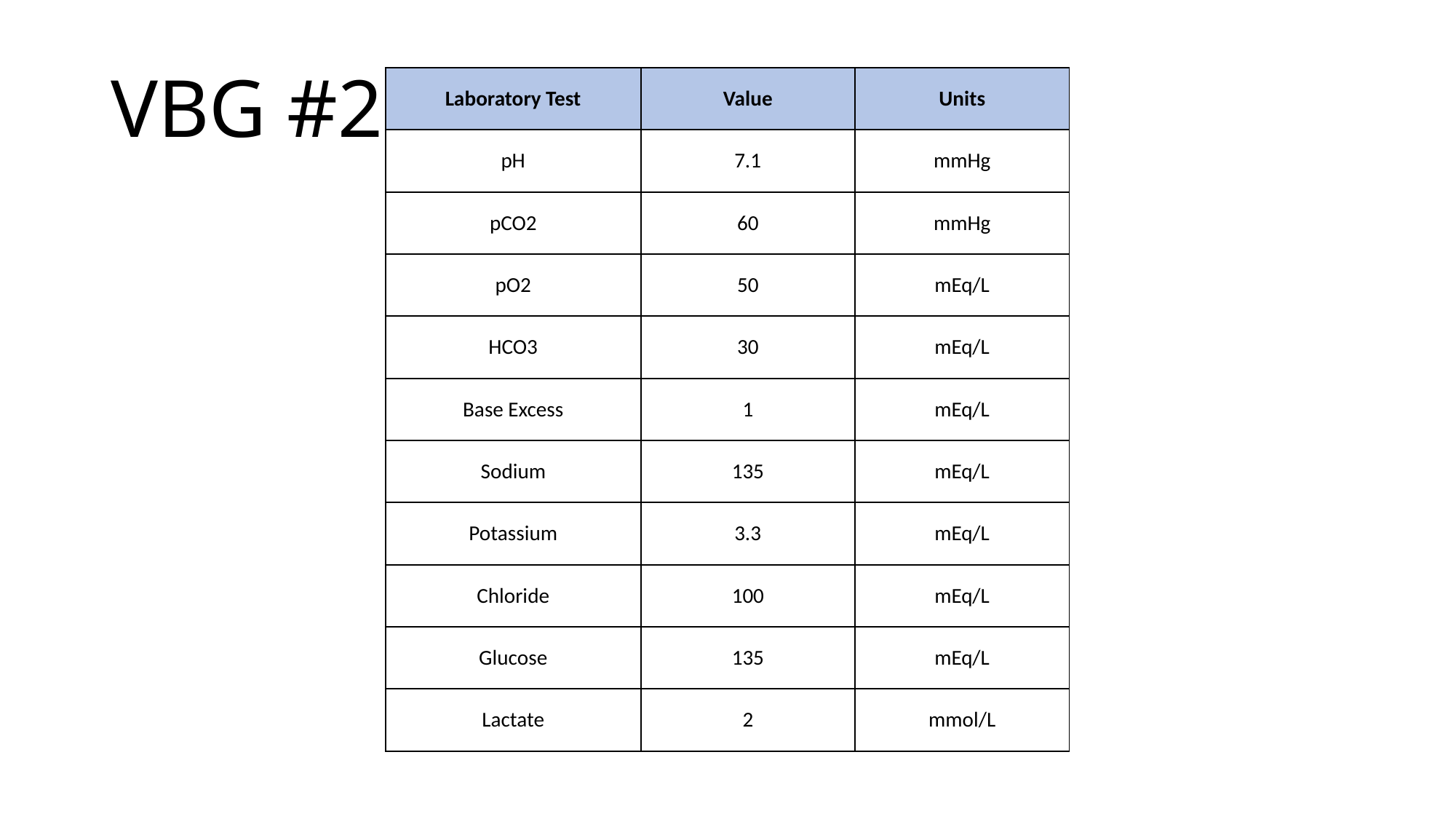

# VBG #2
| Laboratory Test | Value | Units |
| --- | --- | --- |
| pH | 7.1 | mmHg |
| pCO2 | 60 | mmHg |
| pO2 | 50 | mEq/L |
| HCO3 | 30 | mEq/L |
| Base Excess | 1 | mEq/L |
| Sodium | 135 | mEq/L |
| Potassium | 3.3 | mEq/L |
| Chloride | 100 | mEq/L |
| Glucose | 135 | mEq/L |
| Lactate | 2 | mmol/L |

## Slide 8
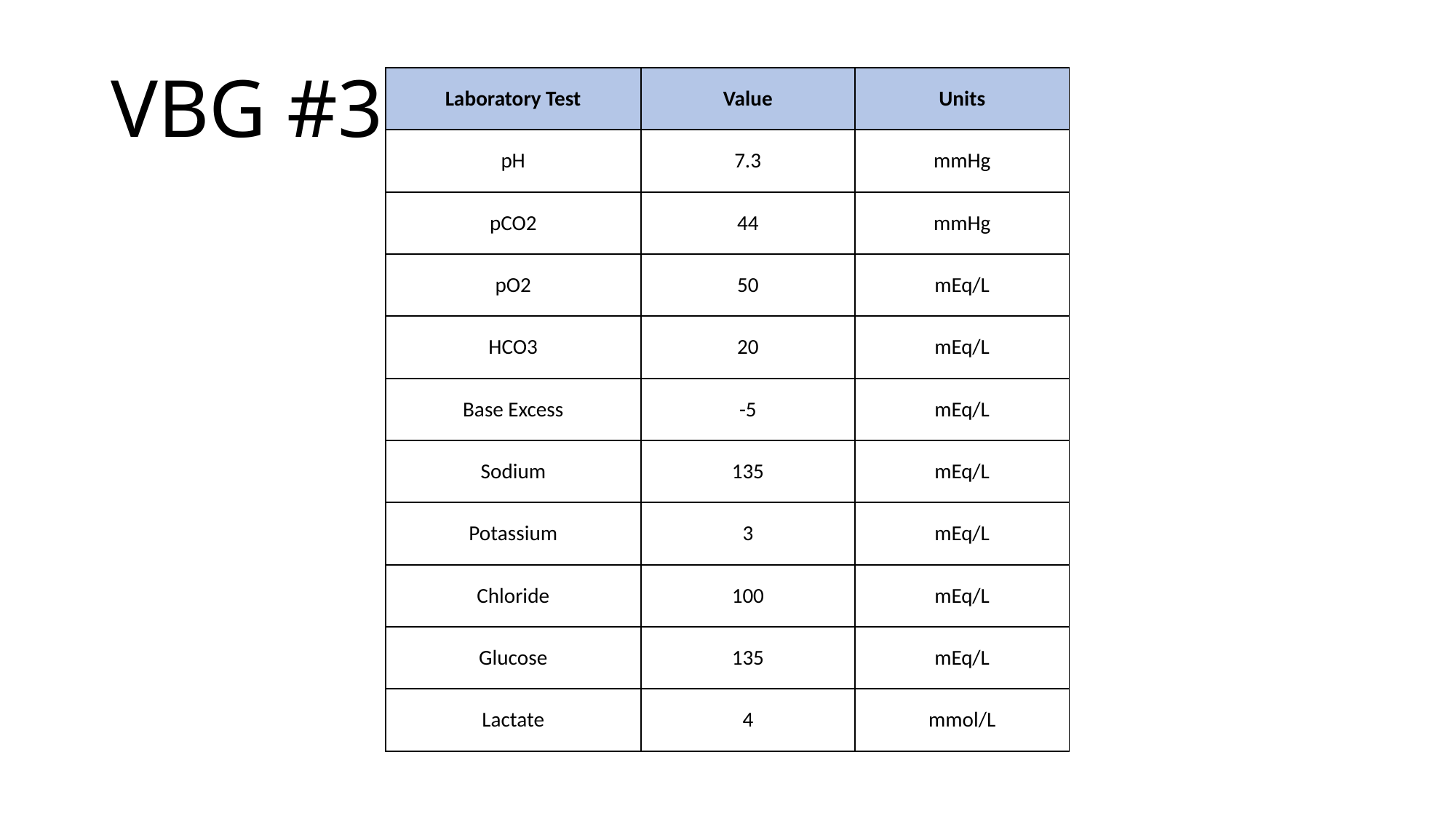

# VBG #3
| Laboratory Test | Value | Units |
| --- | --- | --- |
| pH | 7.3 | mmHg |
| pCO2 | 44 | mmHg |
| pO2 | 50 | mEq/L |
| HCO3 | 20 | mEq/L |
| Base Excess | -5 | mEq/L |
| Sodium | 135 | mEq/L |
| Potassium | 3 | mEq/L |
| Chloride | 100 | mEq/L |
| Glucose | 135 | mEq/L |
| Lactate | 4 | mmol/L |

## Slide 9
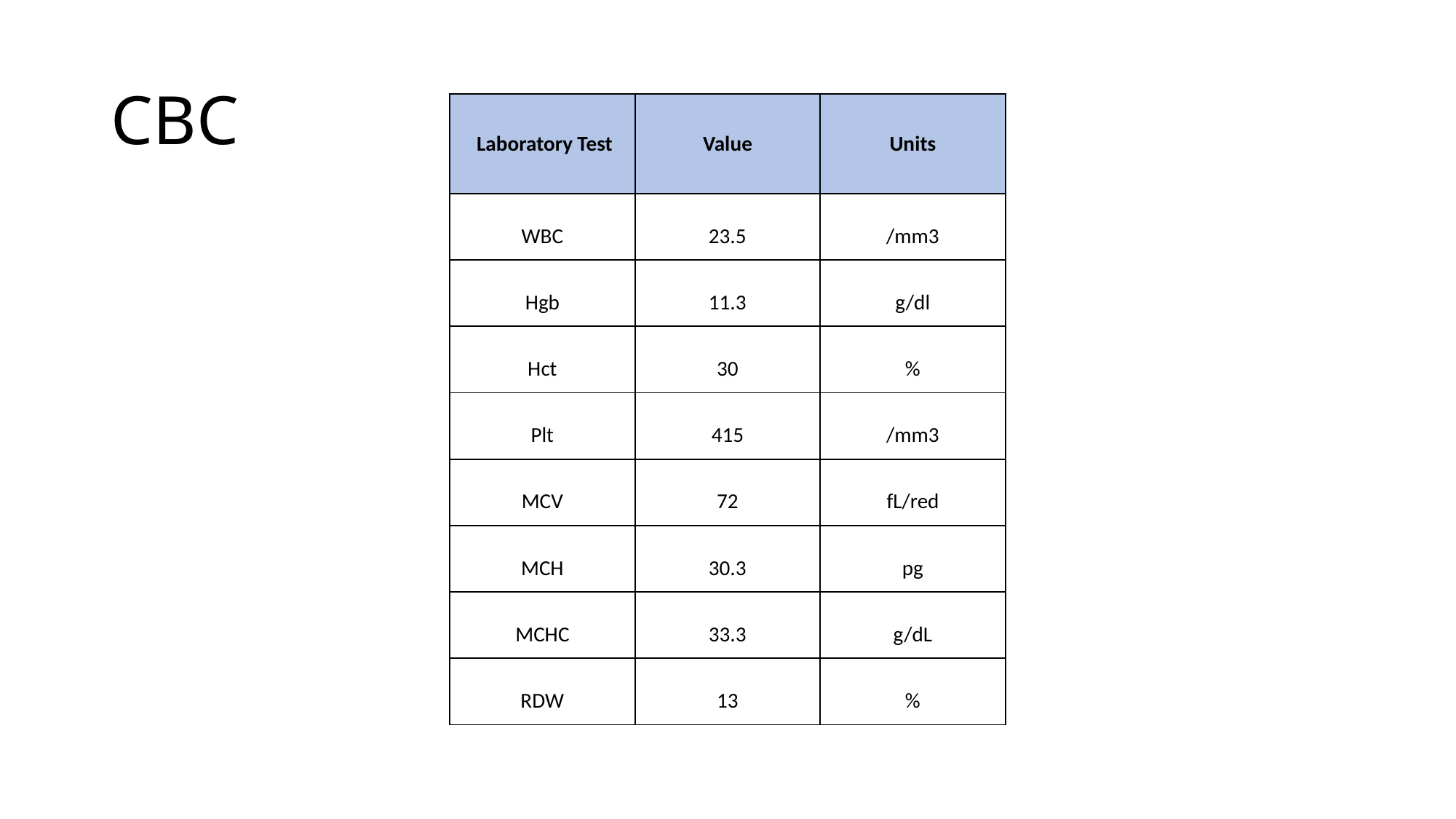

# CBC
| Laboratory Test | Value | Units |
| --- | --- | --- |
| WBC | 23.5 | /mm3 |
| Hgb | 11.3 | g/dl |
| Hct | 30 | % |
| Plt | 415 | /mm3 |
| MCV | 72 | fL/red |
| MCH | 30.3 | pg |
| MCHC | 33.3 | g/dL |
| RDW | 13 | % |

## Slide 10
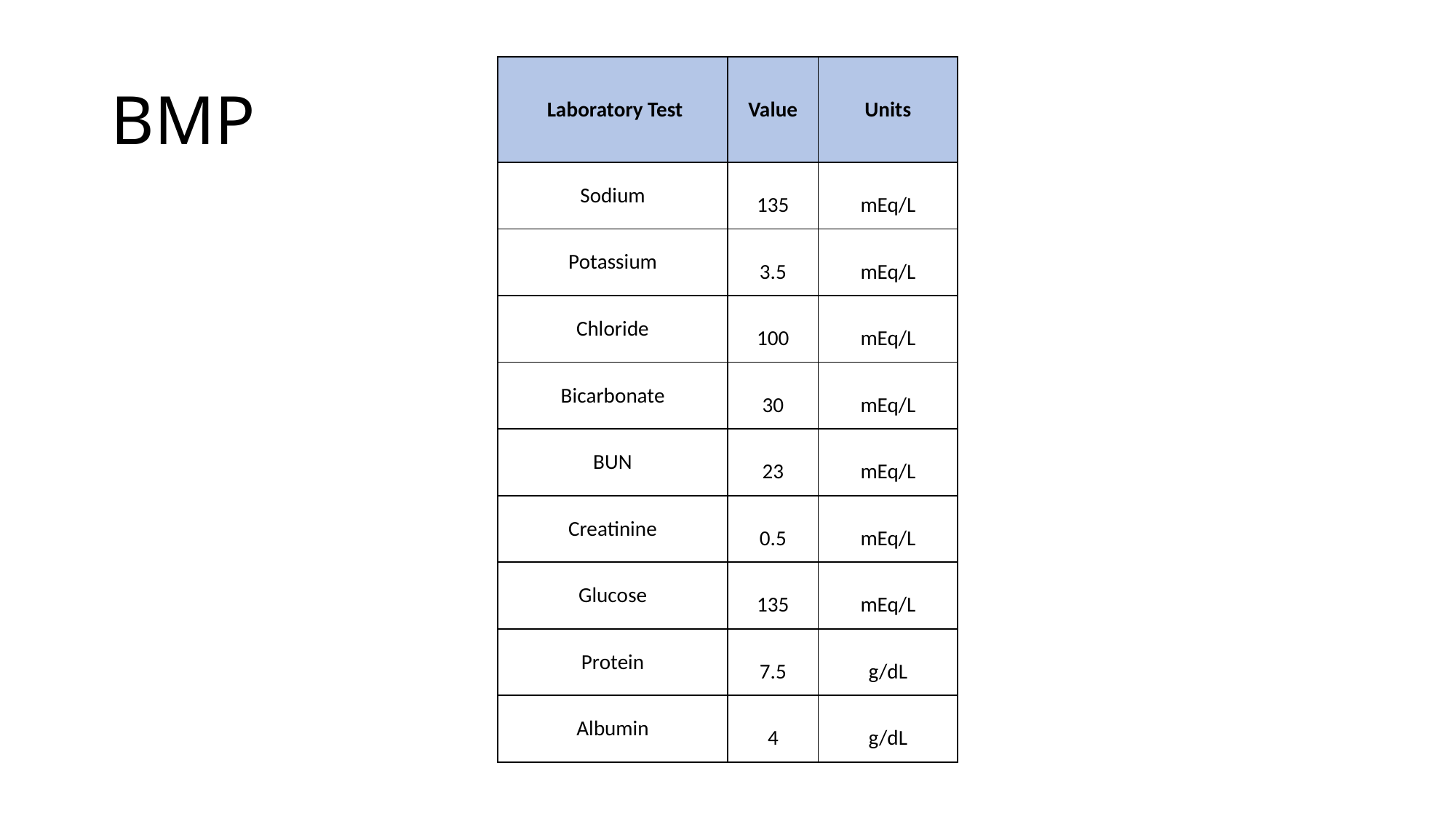

# BMP
| Laboratory Test | Value | Units |
| --- | --- | --- |
| Sodium | 135 | mEq/L |
| Potassium | 3.5 | mEq/L |
| Chloride | 100 | mEq/L |
| Bicarbonate | 30 | mEq/L |
| BUN | 23 | mEq/L |
| Creatinine | 0.5 | mEq/L |
| Glucose | 135 | mEq/L |
| Protein | 7.5 | g/dL |
| Albumin | 4 | g/dL |

## Slide 11
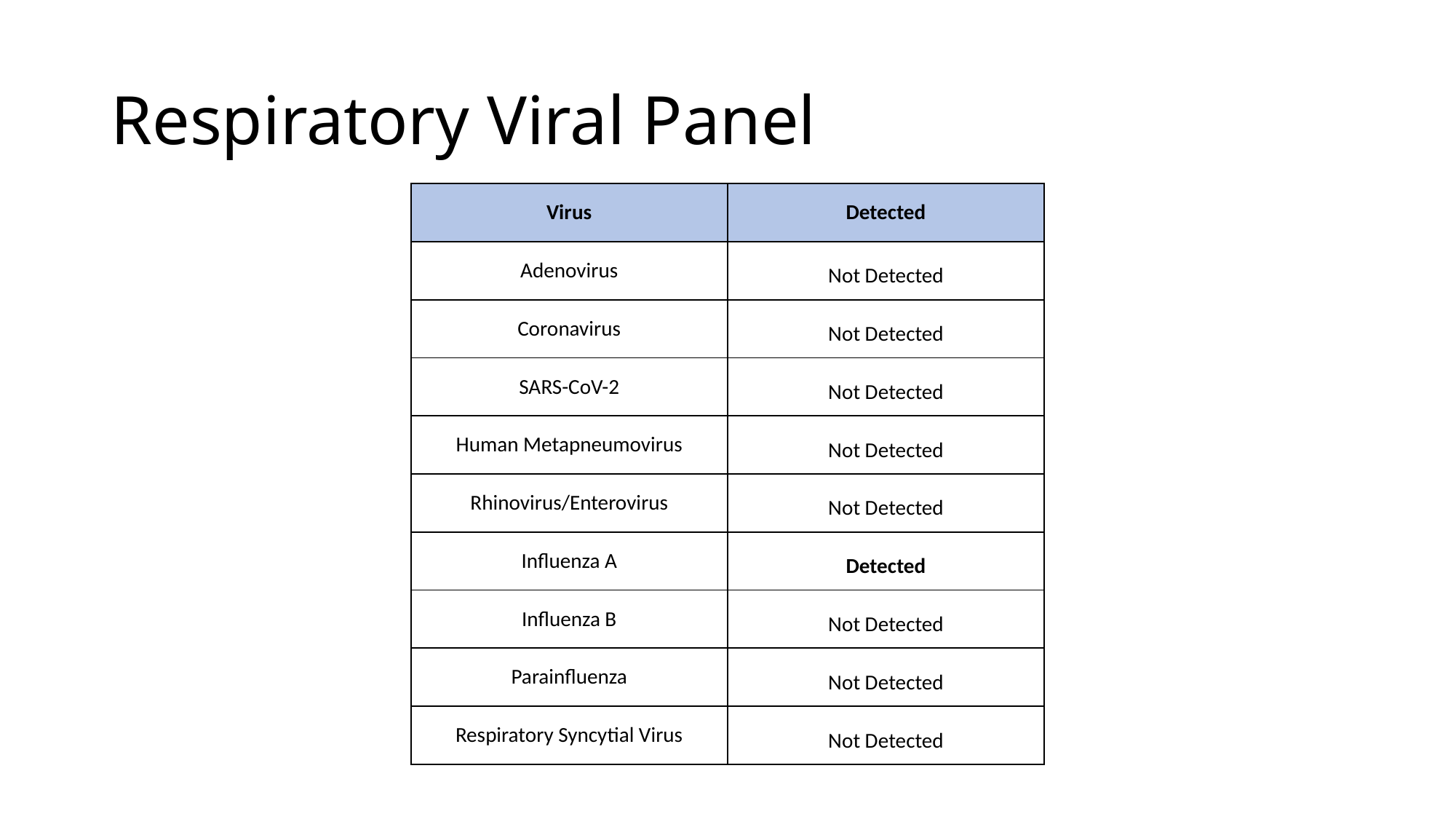

# Respiratory Viral Panel
| Virus | Detected |
| --- | --- |
| Adenovirus | Not Detected |
| Coronavirus | Not Detected |
| SARS-CoV-2 | Not Detected |
| Human Metapneumovirus | Not Detected |
| Rhinovirus/Enterovirus | Not Detected |
| Influenza A | Detected |
| Influenza B | Not Detected |
| Parainfluenza | Not Detected |
| Respiratory Syncytial Virus | Not Detected |

## Slide 12
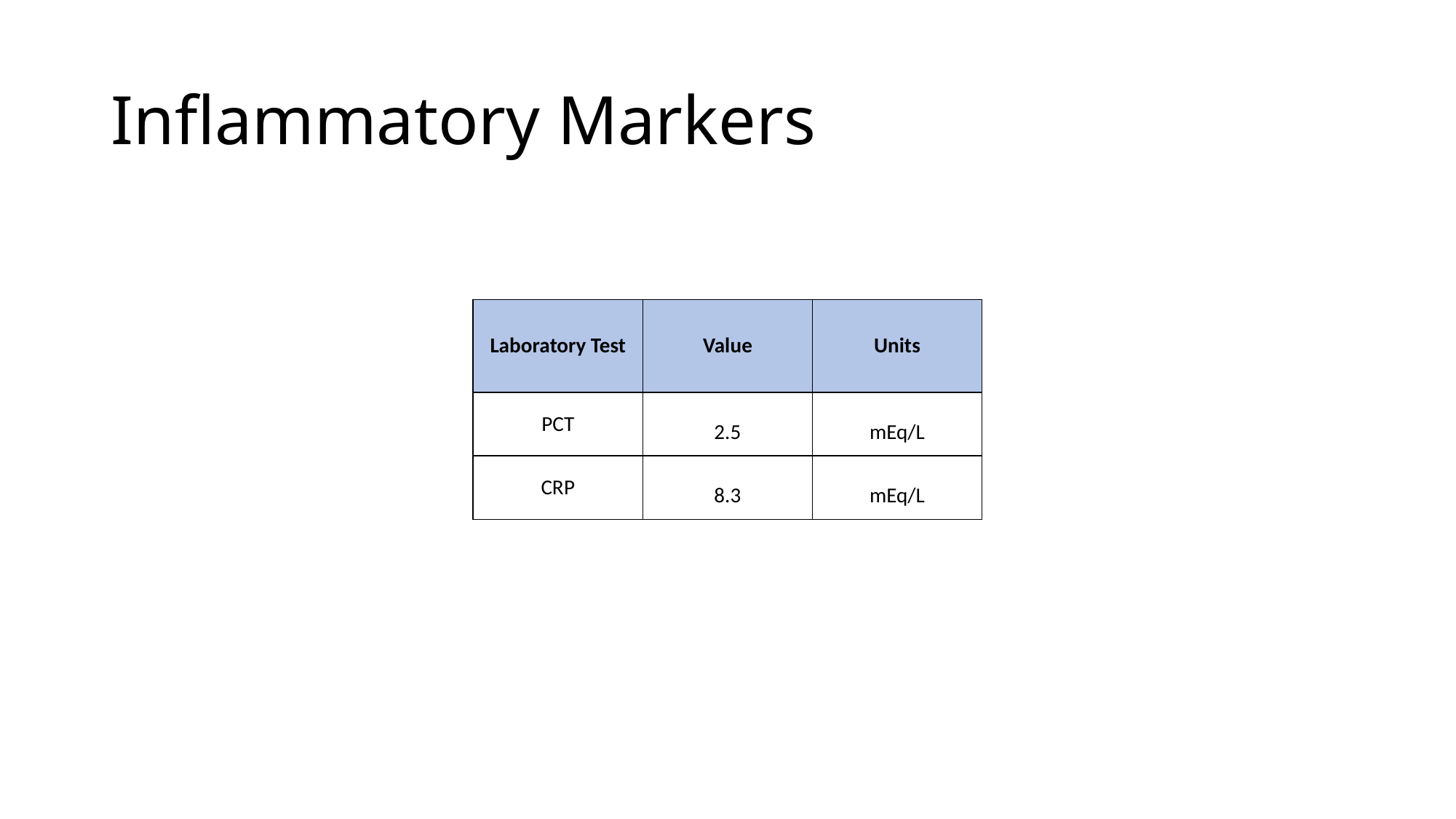

# Inflammatory Markers
| Laboratory Test | Value | Units |
| --- | --- | --- |
| PCT | 2.5 | mEq/L |
| CRP | 8.3 | mEq/L |

## Slide 13
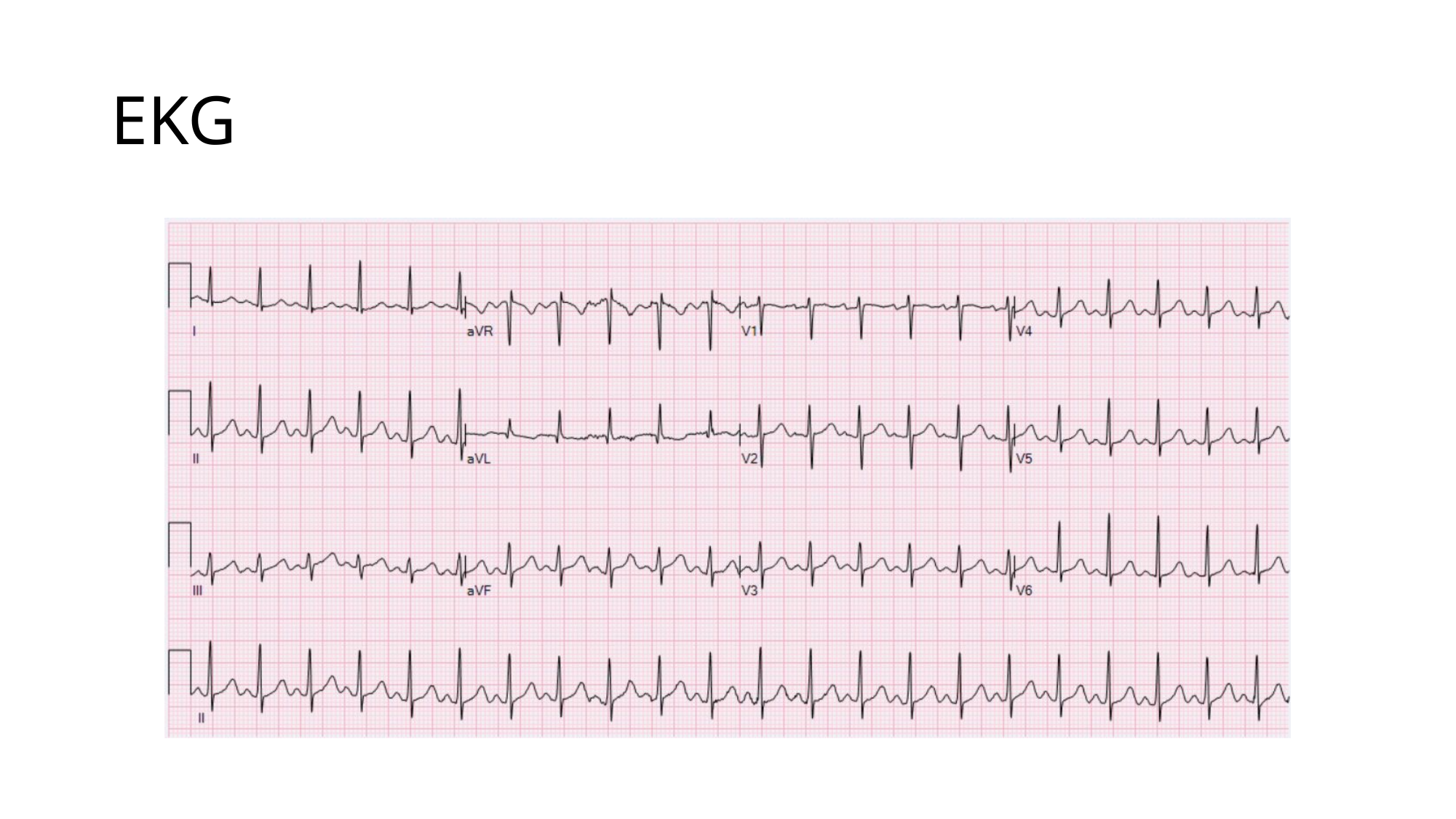

# EKG
