# Supplementary material for: A Simulation-Based Approach to Severe Bronchospasm Complicated by Septic Shock
Source: MedEdPORTAL. 2026 Apr 7;22:11592. doi: 10.15766/mep_2374-8265.11592 (PMC13053521; doi:10.15766/mep_2374-8265.11592)
Supplement: Supplementary file 1 — Simulation Case with Critical Actions.docxSimulation Environmental Preparation List.docxPrebriefing Guide.docxData Slides.pptxDebriefing Guide.docxPostdebrief Handout.docxSimulation Evaluation Form.docx [file mep_2374-8265.11592-s001.zip › E. Debriefing Guide.docx]

**Appendix E. Debriefing Guide**

**SIM De-Briefing Script**

**Phase 1: Introduction**

Thank you all for your participation in our simulation. Now we are going to start our debrief process, which will take about 25 minutes.

Before we get started, I want to repeat some of the main concepts from our pre-briefing discussion. First, we appreciate you immersing yourself in the situation. This is a safe space, and we ask that our discussion and simulation topic remain confidential. We hope that this discussion will give you some learning points to incorporate into your practice in the future. Are we ready to discuss a few points?

**Phase 2: Reactions**

- So, you all just finished a challenging scenario. Could we go around the room and come up with 1-2 words that describe how you are each individually feeling?
- Just to make sure we’re on the same page, let’s talk about what was going on here. Could we have a volunteer (that was not the team leader) tell us what was happening in this scenario?

**Phase 3: Understanding**

After this debrief, I want you all to feel comfortable:

1. Initiating and escalating medication management for bronchospasm.
2. Providing proper respiratory support in a patient with worsening work of breathing.
3. Recognizing shock and identifying the correct vasopressor to use based on a clinical scenario.

I will provide a handout to reinforce these objectives at the end of our session.

Objective 1: Bronchospasm Treatment

- If team correctly provided albuterol, steroids, and magnesium with a bolus:
  - *Preview:* I would like to discuss the team’s management of bronchospasm.
  - *Advocacy*: I saw that you **gave albuterol, steroids, and magnesium with a bolus**.
  - *Advocacy:* I think that escalating to a 2nd agent was appropriate in this patient who was worsening clinically.
  - *Inquiry*: I’m curious what your thought process was here and why you decided to give magnesium instead of another medication?
  - *Listen*: Listen to responses. Consider potential follow up inquiry questions: How do you approach your escalation of medical management for asthma? What medications do you consider? How did you communicate with the pharmacist during escalation (dose, concentration, contraindications, monitoring), and what closed-loop steps did you use to confirm the plan?
    - Teaching point here about asthma medications (use handout).
- If team provided albuterol, steroids, and magnesium without a bolus:
- *Preview:* I would like to discuss the team’s management of bronchospasm.
- *Advocacy*: I saw that you **gave magnesium without a fluid bolus**.
- *Advocacy*: I am concerned that if we give magnesium, it can drop our blood pressure if we don’t give it with a bolus.
- *Inquiry*: I wonder if we could discuss the physiology of Magnesium and its systemic effects.
  - Pearl: Magnesium should always be given with a bolus. Magnesium causes hypotension primarily due to its effects on vascular smooth muscle relaxation and inhibition of calcium-dependent vasoconstriction.
- *Listen*: Listen to responses. Consider potential follow up inquiry questions: How do you approach your escalation of medical management for asthma? What medications do you consider? How did you communicate with the pharmacist during escalation (dose, concentration, contraindications, monitoring), and what closed-loop steps did you use to confirm the plan?
  - Teaching point here about asthma medications (use handout).
- If team provided albuterol and steroids, but did not give magnesium:
  - *Preview:* I would like to discuss the team’s management of bronchospasm.
  - *Advocacy*: I saw that you **did not give magnesium.**
  - *Advocacy*: I worry that in a patient who is that tachypneic and wheezing if we should consider another agent aside from albuterol and steroids alone.
  - *Inquiry*: What were the groups’ thoughts about escalating treatment when this patient worsened from a respiratory perspective?
    - Pearl: Magnesium should always be given with a bolus. Magnesium causes hypotension primarily due to its effects on vascular smooth muscle relaxation and inhibition of calcium-dependent vasoconstriction.
  - *Listen*: Listen to responses. Consider potential follow up inquiry questions: How do you approach your escalation of medical management for asthma? What medications do you consider? How did you communicate with the pharmacist during escalation (dose, concentration, contraindications, monitoring), and what closed-loop steps did you use to confirm the plan?
    - Teaching point here about asthma medications (use handout).

Objective 2: Respiratory Support Escalation

- If team correctly initiated BiPAP:
  - *Preview:* I would like to talk about how you decided on your type of respiratory support for this patient.
  - *Advocacy*: I saw that you started BiPAP on this patient.
  - *Advocacy*: I agree that escalating to BiPAP is appropriate in our patient with near fatal bronchospasm with worsening work of breathing.
  - *Inquiry*: I wonder what your thought process was here when you selected BiPAP over other respiratory modalities, like HHFNC or CPAP?
    - Pearl: BiPAP is preferred pressure support modality for patients with severe asthma.
  - *Listen*: Listen to responses. Consider potential follow up inquiry questions: How does BiPAP help this patient from a pathophysiology perspective?
    - Pearl: BiPAP improves oxygenation via PEEP (EPAP) by keeping airways open + ventilation via IPAP by providing higher inspiratory pressure to improve alveolar ventilation and remove CO_2_ more effectively. It also decreases the work of breathing by providing respiratory support.
    - Teaching point here about respiratory support modalities (use handout).
- If team did not initiate BiPAP:
  - *Preview:* I would like to talk about how you decided on your type of respiratory support for this patient.
  - *Advocacy*: I saw that you started ____ *(anything but BiPAP)* on this patient.
  - *Advocacy*: I worry that escalating not escalating our respiratory support more in a patient with near fatal asthma could worsen his respiratory status.
  - *Inquiry*: I’m curious what other respiratory modalities did you think about starting in this patient and why?
    - Pearl: BiPAP is preferred pressure support modality for patients with severe asthma.
  - *Listen*: Listen to responses. Consider potential follow up inquiry questions: How does BiPAP help this patient from a pathophysiology perspective?
    - Pearl: BiPAP improves oxygenation via PEEP (EPAP) by keeping airways open and ventilation via IPAP by providing higher inspiratory pressure to improve alveolar ventilation and remove CO2 more effectively. It also decreases the work of breathing by assisting respiratory support.
    - Teaching point here about respiratory support modalities (use handout).

Objective 3: Vasopressor Selection

- If team correctly selected norepinephrine or vasopressin:
  - *Preview:* I want to discuss the team’s choice of a pressor in this patient scenario.
  - *Advocacy*: I saw that you started a **norepinephrine** **or vasopressin drip** in this patient.
  - *Advocacy*: I agree that starting a vasopressor given this patient’s severe hypotension with a widened pulse pressure would be beneficial.
  - *Inquiry*: I wonder how you selected the pressor that you chose?
    - Teaching point here about pressor choice (use handout).
  - *Listen:* Listen to responses. Consider potential follow up inquiry questions: This patient had a widened pulse pressure, what could have contributed to a widened pulse pressure in this patient? How did you involve the pharmacist in selecting the vasopressor, and what information could they provide that would change your plan? What closed-loop communication strategies did you use to confirm the medication order, concentration, and titration plan with the pharmacist?
    - Pearl: In this patient, their widened pulse pressure could have been due to: severe asthma (air trapping causing decreased preload and DBP), BiPAP (positive pressure compresses vena cava causing decreased preload and DBP), hypovolemia (decreased preload and resulting lower DBP), and sepsis (increased vascular permeability with drop in DBP).
- If team selected no pressor or a spritzer (1/10^th^ of a code dose):
  - *Preview:* I want to discuss the team’s choice of a pressor in this patient scenario.
  - *Advocacy*: I saw that you **gave a spritzer and/or did not start the patient on a pressor drip**.
  - *Advocacy*: I worry that in a patient with severe hypotension and widened pulse pressure, this patient’s hypotension will continue to worsen without a pressor drip.
  - *Inquiry*: I’m curious to hear what you all discussed when you saw the patient’s blood pressure dropping?
    - Teaching point here about pressor choice (use handout).
  - *Listen:* Listen to responses. Consider potential follow up inquiry questions: This patient had a widened pulse pressure, what could have contributed to a widened pulse pressure in this patient? How did you involve the pharmacist in selecting the vasopressor, and what information could they provide that would change your plan? What closed-loop communication strategies did you use to confirm the medication order, concentration, and titration plan with the pharmacist?
    - Pearl: In this patient, their widened pulse pressure could have been due to: severe asthma (air trapping causing decreased preload and DBP), BiPAP (positive pressure compresses vena cava causing decreased preload and DBP), hypovolemia (decreased preload and resulting lower DBP), and sepsis (increased vascular permeability with drop in DBP).
- If team selected epinephrine:
  - *Preview:* I want to discuss the team’s choice of a pressor in this patient scenario.
  - *Advocacy*: I saw that you **gave Epinephrine.**
  - *Advocacy*: I worry that giving epinephrine to a patient with a HR >190 puts that patient at a high risk of developing a tachyarrhythmia.
  - *Inquiry*: I wonder if you can discuss downside effects of epinephrine in the setting of tachycardia?
    - Pearl: Given epinephrine’s beta-1 agonism, we can have worsening tachycardia and potentially send the patient into a tachyarrhythmia.
    - Teaching point here about pressor choice (use handout).
  - *Listen:* Listen to responses. Consider potential follow up inquiry questions: This patient had a widened pulse pressure, what could have contributed to a widened pulse pressure in this patient? How did you involve the pharmacist in selecting the vasopressor, and what information could they provide that would change your plan? What closed-loop communication strategies did you use to confirm the medication order, concentration, and titration plan with the pharmacist?
    - Pearl: In this patient, their widened pulse pressure could have been due to: severe asthma (air trapping causing decreased preload and DBP), BiPAP (positive pressure compresses vena cava causing decreased preload and DBP), hypovolemia (decreased preload and resulting lower DBP), and sepsis (increased vascular permeability with drop in DBP).

**Phase 4: Summary**

I’d like to wrap up the debriefing now. Can you tell me one takeaway point that you’ll incorporate into your practice in the future?

Here is a handout that can provide more information about our 3 learning objectives:

1. An **asthma medication table** to help with initiating and escalating medication management for bronchospasm.
2. A **respiratory support char**t to reference in a patient with worsening bronchospasm who you are trying to identify which modality to use.
3. A comparison of the **major vasopressors** when selecting a pressor for a patient with shock.

Now that we are finished with the debriefing, I would appreciate it if each of you could fill out a brief 1-minute survey to assess your learning and understanding of the objectives. Please circle the box that most applies to you.
