# Supplementary material for: A Simulation-Based Approach to Severe Bronchospasm Complicated by Septic Shock
Source: MedEdPORTAL. 2026 Apr 7;22:11592. doi: 10.15766/mep_2374-8265.11592 (PMC13053521; doi:10.15766/mep_2374-8265.11592)
Supplement: Supplementary file 1 — Simulation Case with Critical Actions.docxSimulation Environmental Preparation List.docxPrebriefing Guide.docxData Slides.pptxDebriefing Guide.docxPostdebrief Handout.docxSimulation Evaluation Form.docx [file mep_2374-8265.11592-s001.zip › F. Postdebrief Handout.docx]

**Appendix F. Postdebrief Handout**

**Asthma Medications Dosing & Mechanism**

| **Drug** | **Mechanism** | **Dosing** |
| --- | --- | --- |
| Albuterol | Bronchodilator, Beta-2 agonist | 5mg if >10kg, 2.5mg if <10kg |
| Dexamethasone | Steroid, anti-inflammatory | 0.6mg/kg |
| Prednisolone | Steroid, anti-inflammatory | 1-2mg/kg/day divided BID |
| Methylprednisolone | Steroid, anti-inflammatory | 1mg/kg q6h |
| Ipratropium | Bronchodilator, anticholinergic | 500mcg if >10kg, 250mcg if <10kg |
| Magnesium | Smooth muscle relaxant | 50mg/kg up to 2g IV |
| Terbutaline | Bronchodilator, Beta-2 agonist | Loading dose followed by drip |
| Theophylline/  Aminophylline | Bronchodilator, phosphodiesterase inhibitor, increasing the levels of cAMP | Loading dose followed by drip |
| Epinephrine | Bronchodilator, Beta-2 agonist | IM 0.01 mg/kg (~0.3-0.5 mg) |

**Respiratory Support Modalities**

| **Mode** | **FiO₂** | **Flow Rate** | **Indications** | **Mechanism** | **Advantages** | **Limitations** |
| --- | --- | --- | --- | --- | --- | --- |
| NC | 24-44% | 1-6 L/min | Mild hypoxia | Low-flow oxygen | Well tolerated, allows eating/talking | Limited FiO₂, ineffective for WOB |
| VM | 24-50% | 5-15 L/min (varies by adapter) | Controlled oxygen delivery needs | Mixes oxygen with room air for precise FiO₂ delivery | Provides accurate FiO₂, good for CO₂ retainers | Limited FiO₂, not suitable for severe hypoxia |
| NRB | 60-90% | 10-15 L/min | Severe hypoxia, pre-intubation, shock | Reservoir bag provides high FiO₂, prevents rebreathing CO₂ | High FiO₂, easy to use | No ventilation support, risk of CO₂ retention if not at high flow |
| HHFNC | Up to 100% | 1-2 L/kg/min (max 60) | Moderate hypoxia, bronchiolitis, pneumonia, early respiratory distress | Provides heated, humidified high-flow oxygen; reduces dead space and may generate low-level PEEP | Improved oxygenation & CO₂ clearance, reduces work of breathing | Can cause air trapping, gastric distension |
| CPAP | 40-100% | Variable | Moderate to severe respiratory distress, apnea | Provides continuous airway pressure (PEEP) to keep alveoli open | Prevents atelectasis, reduces WOB, improves oxygenation | Patient must breathe spontaneously, risk of pneumothorax |
| BiPAP | 40-100% | Variable | Respiratory failure, severe asthma, neuromuscular weakness | Alternates insp. (IPAP) & exp. (EPAP) pressures to assist ventilation | Reduces CO₂, improves ventilation & oxygenation | Risk of air trapping, gastric insufflation |

**Septic Shock: Pressors**

| **Vasopressor** | **Indications** | **Mechanism** |
| --- | --- | --- |
| Epinephrine | - Cold Shock (low CO, ↑ SVR) - HR normal/slightly high - Weak pulses - Delayed cap refill - Narrow pulse pressure (<40mmHg) | - Alpha-1 agonist: Vasoconstriction - Beta-1 agonist: Increases HR & contractility - Beta-2 agonist |
| Norepinephrine | - Warm Shock (↑ CO, low SVR) - Increased HR - Bounding pulses - Flash cap refill - Wide pulse pressure | - Alpha-1 agonist: Strong vasoconstriction - Beta-1 agonist: Mild increase HR & contractility (*less than epinephrine*) |
| Vasopressin | - Refractory septic shock unresponsive to catecholamines - Severe vasodilatory shock with low MAP despite other pressors | - V1 receptor agonist: Vasoconstriction independent of catecholamines |
| Dopamine | - Vasopressor in fluid-refractory hypotension - May be less effective than epinephrine/norepinephrine, no longer considered first-line | - Low dose: Dopaminergic (renal vasodilation) - Moderate dose: Beta-1 agonist (increases HR & contractility) - High dose: Alpha-1 agonist (vasoconstriction) |
| Dobutamine | - Myocardial dysfunction with low EF, poor contractility - Cold shock with low CO despite epinephrine | - Beta-1 agonist: Increases contractility & HR - Mild Beta-2 agonist: Mild vasodilation |
| Milrinone | - Low CO with high SVR - Myocardial dysfunction with pulmonary hypertension | - PDE-3 inhibitor: Increases cAMP → Enhances contractility & vasodilation |
