# Supplementary material for: A Simulation-Based Approach to Severe Bronchospasm Complicated by Septic Shock
Source: MedEdPORTAL. 2026 Apr 7;22:11592. doi: 10.15766/mep_2374-8265.11592 (PMC13053521; doi:10.15766/mep_2374-8265.11592)
Supplement: Supplementary file 1 — Simulation Case with Critical Actions.docxSimulation Environmental Preparation List.docxPrebriefing Guide.docxData Slides.pptxDebriefing Guide.docxPostdebrief Handout.docxSimulation Evaluation Form.docx [file mep_2374-8265.11592-s001.zip › G. Simulation Evaluation Form.docx]

**Appendix G. Simulation Evaluation Form**

**Aaron Huffman: A Child with Respiratory Distress**

**Instructor: Date:**

**What type of resident (please circle one on each line):**

Peds Peds Neuro Triple Board IM/Peds Pharmacy

PGY-1 PGY-2 PGY-3 PGY-4

|  | **Yes** | **No** |
| --- | --- | --- |
| This case presented during the simulation is relevant to my work. |  |  |
| The simulation case was realistic. |  |  |
| The debrief promoted reflection and team discussion. |  |  |
| The group discussion helped me develop and prioritize evaluation and management options for a child with bronchospasm. |  |  |
| The facilitators created a safe environment for discussion and exploration. |  |  |

**AFTER** participating in this session, how confident are you in your ability to:

|  | **1 Needs Improvement** | **2 Proficient** | **3 Mastery** |
| --- | --- | --- | --- |
| Identify and treat bronchospasm appropriately. | Identify and treat bronchospasm with **first** **line** management | Identify and treat bronchospasm with **first- and second-line** management | Identify and treat near fatal bronchospasm with a **range of modalities** |
| Escalate respiratory support for a patient with near fatal bronchospasm. | Identify need for escalation but unsure which modality to use | Identify need for escalation to include **positive pressure** | Escalate to **BiPAP** appropriately |
| Recognize severe hypotension and identify the correct pressor based on a patient’s clinical presentation. | Recognize shock and give **fluids only** | Recognize shock and identify **need for a pressor** | Recognize shock and identify **correct pressor** |

**BEFORE** participating in this session, how confident are you in your ability to:

|  | **1 Needs Improvement** | **2 Proficient** | **3 Mastery** |
| --- | --- | --- | --- |
| Identify and treat bronchospasm appropriately. | Identify and treat bronchospasm with **first** **line** management | Identify and treat bronchospasm with **first- and second-line** management | Identify and treat near fatal bronchospasm with a **range of modalities** |
| Escalate respiratory support for a patient with near fatal bronchospasm. | Identify need for escalation but unsure which modality to use | Identify need for escalation to include **positive pressure** | Escalate to **BiPAP** appropriately |
| Recognize severe hypotension and identify the correct pressor based on a patient’s clinical presentation. | Recognize shock and give **fluids only** | Recognize shock and identify **need for a pressor** | Recognize shock and identify **correct pressor** |

How can we improve this simulation?
