# Supplementary material for: A Simulation-Based Approach to Severe Bronchospasm Complicated by Septic Shock
Source: MedEdPORTAL. 2026 Apr 7;22:11592. doi: 10.15766/mep_2374-8265.11592 (PMC13053521; doi:10.15766/mep_2374-8265.11592)
Supplement: Supplementary file 1 — Simulation Case with Critical Actions.docxSimulation Environmental Preparation List.docxPrebriefing Guide.docxData Slides.pptxDebriefing Guide.docxPostdebrief Handout.docxSimulation Evaluation Form.docx [file mep_2374-8265.11592-s001.zip › _Educational Summary Report_11592.pdf]

MedEdPORTAL
THE AAMC JOURNAL OF TEACHING AND LEARNING RESOURCES

Original Publication
Open Access

A Simulation-Based Approach to Severe Bronchospasm Complicated by Septic Shock

Taylor Anne Merritt, MD*, Annesha Dutta, MD, Yarah Ghotmi, MD, Ngoc Van Horn, MD

*Corresponding author: Taylor.Merritt@UTSouthwestern.edu

Abstract

Introduction: Recognizing and managing high-acuity, low-frequency pediatric conditions such as near-fatal bronchospasm with respiratory failure and uncompensated septic shock are essential skills for trainees. These emergencies require timely clinical judgment and familiarity with medications, respiratory support, and resuscitation strategies. **Methods:** We implemented a high-fidelity, interprofessional simulation for pediatric residents focused on management of near-fatal bronchospasm complicated by septic shock. The simulation was delivered as 4 standalone sessions over 2 months. The scenario features a 2-year-old child presenting in respiratory distress due to pneumonia, who progresses to respiratory failure requiring BiPAP. He subsequently develops uncompensated septic shock necessitating fluid resuscitation and vasopressor support. Simulation materials included a structured prebrief, PEARLS-guided debrief, and a retrospective post-then-presimulation confidence survey. **Results:** Among 90 pediatric residents (32 PGY 1, 34 PGY 2, and 24 PGY 3/4), confidence scores significantly improved across all 3 objectives (each $P < .01$). For objectives 1, 2, and 3, perceived confidence improved at least one level in 62%, 67%, and 72% of residents, respectively. PGY-1 residents showed the most improvement overall, with significantly greater confidence gains compared with senior residents in implementing objective 2 (respiratory support escalation) ($P = .004$). **Discussion:** Following a high-fidelity simulation focused on management of near-fatal bronchospasm, respiratory failure, and uncompensated septic shock, pediatric residents reported increased confidence across all training levels in achieving the educational objectives. These outcomes align with Kirkpatrick Level 2 learning, supporting simulation as a valuable tool to enhance clinical confidence in recognizing and treating rare but life-threatening scenarios without patient risk.

Keywords
Competency-Based Medical Education, Pediatrics, Simulation, Bronchospasm, Septic Shock, High-Fidelity, Interprofessional

Educational Objectives

By the end of this activity, learners will report increased confidence in:

1. Identifying and treating a patient with near-fatal bronchospasm.
2. Creating an appropriate respiratory support plan for a patient with progression to respiratory failure.
3. Choosing appropriate resuscitation interventions for septic shock.

Citation:
Merritt TA, Dutta A, Ghotmi Y, Van Horn N. A simulation-based approach to severe bronchospasm complicated by septic shock. *MedEdPORTAL*. 2026;22:11592.
https://doi.org/10.15766/mep_2374-8265.11592

Introduction

Near-fatal bronchospasm is a severe, life-threatening constriction of the smooth muscles in the bronchial walls, causing airflow obstruction. This constriction can lead to respiratory failure if not rapidly recognized and treated. Severe bronchospasm remains a leading cause of pediatric emergency department visits and hospitalizations. Asthma, one of the primary drivers of bronchospasm, accounts for over 270,000 pediatric emergency department visits annually, with 10% of patients requiring an inpatient stay.[1] While most children respond to initial therapies, up to 2% of patients progress to near-fatal bronchospasm and respiratory failure, particularly when complicated by lower respiratory infection.[2]

Lower respiratory infections are a well-recognized driver of pediatric bronchospasm, and when untreated, can result in sepsis and septic shock. Pediatric sepsis is characterized by infection with associated organ dysfunction. When severe, sepsis

Copyright © 2026 Merritt et al. This is an open-access publication distributed under the terms of the Creative Commons Attribution license.

may evolve into septic shock, with resulting cardiovascular dysfunction such as hypotension or the need for vasoactive support. Among children with infection, nearly 7% meet sepsis criteria, and when this progresses to septic shock, mortality rates can range from 10% to 33.5% depending on the setting.[3] Evidence demonstrates that early detection and timely treatment with fluid resuscitation and antimicrobials can significantly reduce morbidity and mortality.[4] Despite the clinical urgency of these conditions, pediatric trainees frequently report limited confidence in managing these emergent conditions, particularly based on clinical exposure alone.[5,6]

Simulation-based education has emerged as a powerful tool to address this gap and has demonstrated superior outcomes compared with those achieved by traditional clinical education.[7] High-fidelity simulation improves not only technical performance but also crisis resource management and interprofessional communication in pediatric resuscitation settings.[8,9] Specific studies have shown that simulation training increases adherence to sepsis bundles, enhances team communication during resuscitation, and improves learner confidence in managing respiratory failure.[10,11] Simulation is therefore well-suited for high-acuity, low frequency (HALF) scenarios where real-world practice opportunities are limited, but clinical consequences are severe if management is delayed or inadequate.

However, the broader literature on pediatric simulation often focuses on single-system emergencies rather than the complex interplay of multiple, rapidly evolving conditions.[8-11] Most available curricula are largely organized around discrete single-system emergencies, such as respiratory distress or status asthmaticus, or around isolated shock resuscitation scenarios, including pediatric sepsis and septic shock resuscitation, rather than integrated, evolving multisystem deterioration.[12-15] Few published curricula require learners to navigate a trajectory that progresses from near-fatal bronchospasm to septic shock, forcing prioritization of airway management, ventilatory support, and hemodynamic resuscitation.

This gap underscores the need for simulation-based educational resources that reflect the clinical reality of pediatric care, where patients frequently present with evolving physiology and multiple concurrent problems rather than isolated diagnoses. Published simulation cases demonstrate that learners find evolving, multistep scenarios realistic and educationally valuable; for example, a reported emergency simulation in which a patient progresses from leukemia blast crisis to respiratory failure and cardiac arrest was rated as both realistic and useful by most participants.[16] Although direct comparisons between complex, evolving cases and separate, single-diagnosis simulations are limited in the literature, we selected this approach intentionally to mirror real-world clinical trajectories. Additionally, the use of a single integrated case was pragmatic, given resident scheduling constraints, and allowed exposure to multiple illness scripts and management decisions within a single educational session.

To address this gap in the literature, we developed and implemented a high-fidelity simulation case designed to improve pediatric residents' confidence in managing near-fatal bronchospasm complicated by septic shock.

Methods

Development

We developed this simulation with a multidisciplinary team, adapting a real clinical case to maximize educational value. Our faculty simulation director, a pediatric emergency medicine physician and instructional design specialist, oversaw scenario development, revision, and implementation. Simulation operations staff and a simulation pharmacy supervisor reviewed the case for clinical feasibility and medication safety, and 2 pediatric colleagues provided independent peer review.

The simulation was developed in alignment with best practice guidelines from the Society for Simulation in Healthcare and integrated key elements of Zone 3 simulation, including interdisciplinary teamwork, real-world clinical distractions, and a multiphase scenario structure.[17] The simulation was mapped to core Entrustable Professional Activities (EPAs) to ensure alignment with pediatric residency competencies.

This simulation was performed in the simulation laboratory at the University of Texas Southwestern medical school as part of the pediatric residency curriculum (Appendix A). All pediatric residents were assigned to 1 of 4 scheduled sessions and then subdivided into smaller groups for participation. Learners included categorical pediatric residents, as well as those from combined programs—medicine-pediatrics, pediatric neurology, and triple board (pediatrics, general psychiatry, and child/adolescent psychiatry)—in addition to pharmacy residents. The learners were not provided any learning materials prior to the scenario.

Equipment/Environment

We recommend the following equipment for successful implementation of this case, with the complete list in Appendix B:

- High-fidelity manikin that can display evolving physical examination findings, including wheezing and poor perfusion (Laerdal Pedi-HAL)

Copyright © 2026 Merritt et al. This is an open-access publication distributed under the terms of the Creative Commons Attribution license.

- Noninvasive blood pressure cuff, pulse oximeter, and heart rate leads attached to the manikin
- Pediatric respiratory support equipment, including oxygen hook up, bag-valve mask, non-rebreather, nasal cannula, high-flow nasal cannula, and bilevel positive airway pressure (BiPAP) mask
- Monitor to display the patient's vital signs, including blood pressure, heart rate, respiratory rate, oxygen saturation, and temperature
- Appropriate medications with syringes and needles of various sizes, including acetaminophen, ceftriaxone, vancomycin, norepinephrine, epinephrine, normal saline, albuterol, ipratropium, magnesium sulfate, solumedrol, prednisolone, and dexamethasone
- Video screen to display laboratory results and imaging
- Paperwork from outside hospital including emergency department discharge summary and chest radiograph

Personnel

In the clinical scenario, the learners included 4–5 pediatric residents and 1 pharmacy resident. The primary facilitator was a pediatric trainee or faculty member trained in best practice for simulation facilitation. The primary facilitator provided additional pertinent information as needed based on learner request. A pharmacy attending was also available during the simulation and for the debrief to provide additional instruction regarding medication management. A simulation technician was responsible for changing vital signs and manikin physical examination during the simulation.

Implementation

Prior to the simulation, the facilitator presented the learners with a 10-minute prebrief that included an introduction of team members, goals of the activity, timeline, basic assumption of simulation, fiction contract, confidentiality, safety, and roles (Appendix C). The learners were then oriented to the case and given the prompt. Before entering the simulation room, the facilitator designated a team leader from the medical residents, who then assigned roles to the rest of the team. The pharmacy resident served as the pharmacist, and the remaining medical residents assumed physician roles as assigned by the team leader (e.g., airway management, physical examination, medication administration), though they were all expected to contribute to medical decision making.

The facilitator then entered a separate room with a one-way mirror where the facilitator, pharmacist, and simulation technician were able to view and hear the learners. The facilitator used an intercom to communicate relevant information as needed. The primary facilitator evaluated the participants by checking off the critical actions on the critical action flowsheet. The simulation began with a custom AI-generated, virtual, embedded simulation participant that acted as the emergency medical services (EMS) worker who provided handoff on the patient from the outside emergency department (Appendix D, video 1). Discharge paperwork from the outside emergency department was also available in the room (Appendix D). A video of a child in moderate respiratory distress was shown on the monitor at the start of the case (Appendix D, video 2). Use of the virtual embedded simulation participant and respiratory distress videos are optional; alternatively, the facilitator may provide the handoff from the EMS worker and describe the child's work of breathing when requested by learners. All laboratory results and imaging were displayed on a screen in the room as requested by learners (Appendix D). The simulation took approximately 15 minutes to complete. The case is fully presented with a critical action flowsheet in Appendix A.

Debriefing

The debriefing lasted 25 minutes and followed the Promoting Excellence and Reflective Learning in Simulation (PEARLS) model.[18] All facilitators were evaluated with Debriefing Assessment for Simulation in Healthcare (DASH) to ensure high-quality debriefing to cover the necessary educational objectives. Each session was co-facilitated by a medical facilitator and pharmacy facilitator, with both medical and pharmacy residents participating in the debrief. The primary facilitator began the debrief by thanking the participants and reviewing the concepts of fiction contract, safety, and confidentiality. Each participant was asked to provide 1 word to describe their feelings postsimulation. After acknowledging these initial feelings, 1 participant was asked to provide a summary of the case. Once a shared case summary was agreed upon by all participants, the 3 specific educational objectives (bronchospasm treatment, respiratory support management, and vasopressor selection) were reviewed, with a focus on interprofessional communication between medical and pharmacy residents. At the end of the debrief, medical residents were asked to stay to complete a standardized survey assessment, while pharmacy residents returned to the simulation room with the pharmacy attending to review any specific medication questions.

Each educational objective was debriefed separately in the analysis phase, utilizing PAAIL (Preview, Advocacy, Inquiry, Listen) educational strategies based on the critical action flowsheet, with emphasis on 1–2 clinical conclusions (knowledge pearls) per educational objective (Appendix E). The facilitator

Copyright © 2026 Merritt et al. This is an open-access publication distributed under the terms of the Creative Commons Attribution license.

then summarized by asking each learner for 1 key take-away before reviewing the 3 objectives again, after distributing the educational handout (Appendix F).

The following are examples the facilitator used during the debrief for educational objective 1 utilizing the PAAIL strategy for debriefing:

- *Preview:* I would like to discuss the team's management of bronchospasm.
- *Advocacy*: I saw that you gave albuterol, steroids, and magnesium with a bolus.
- *Advocacy:* I think that escalating to a second agent was appropriate in this patient who was worsening clinically.
- *Inquiry*: I'm curious what your thought process was here and why you decided to give magnesium instead of another medication?
- *Listen*: Listen to responses. Consider potential follow up inquiry questions: How do you approach your escalation of medical management for asthma? What medications do you consider? How did you communicate with the pharmacist during escalation (dose, concentration, contraindications, monitoring), and what closed-loop steps did you use to confirm the plan?

The following are examples the facilitator used during the debrief of educational objective 2 utilizing the advocacy-inquiry strategy:

- *Preview:* I would like to talk about how you decided on your type of respiratory support for this patient.
- *Advocacy*: I saw that you started BiPAP on this patient.
- *Advocacy*: I agree that escalating to BiPAP is appropriate in our patient with near fatal bronchospasm with worsening work of breathing and hypercarbia.
- *Inquiry*: I wonder what your thought process was here when you selected BiPAP over other respiratory modalities, like high flow or CPAP?
- *Listen*: Listen to responses. Consider potential follow up inquiry questions: How does BiPAP help this patient from a pathophysiology perspective?

The following are examples the facilitator used during the debrief of educational objective 3 utilizing the advocacy-inquiry strategy:

- *Preview:* I want to discuss the team's choice of a pressor in this patient scenario.
- *Advocacy:* I saw that you started a norepinephrine drip in this patient.
- *Advocacy:* I agree that starting a vasopressor given this patient's severe hypotension with a widened pulse pressure would be beneficial.
- *Inquiry:* I wonder how you selected the pressor that you chose?
- *Listen:* Listen to responses. Consider potential follow up inquiry questions: This patient had a widened pulse pressure, what could have contributed to a widened pulse pressure in this patient? How did you involve the pharmacist in selecting the vasopressor, and what information could they provide that would change your plan? What closed-loop communication strategies did you use to confirm the medication order, concentration, and titration plan with the pharmacist?

Assessment

During the simulation, participants were assessed using the critical action flowsheet checklist (Appendix A). The checklist identifies 3 critical actions, each aligned with an educational objective, that teams must complete to progress to the next frame. If a team missed a critical action, facilitators provided prompts (or "lifesavers") designed to redirect them toward the corresponding objective. Learners then received structured feedback based on the checklist after the case during the debriefing.

Following the debrief, medical resident participants completed a single combined survey using a retrospective post-then-presimulation format to rate their confidence in achieving the educational objectives after the simulation and, using the same frame of reference, to reflect and rate their confidence before the simulation (Appendix G). We selected this approach because simulation can recalibrate learners' understanding of the clinical tasks and decisions required by the objectives; collecting both ratings after the session reduces response-shift bias and supports more comparable presimulation and postsimulation self-efficacy ratings.[19] This design also reduces administrative burden and missing data by capturing both time points in 1 instrument. The University of Texas Southwestern Institutional Review Board reviewed this project (IRB no. STU20250525) and determined that it did not require a coverage analysis or further IRB oversight as human subject research.

Participation in the survey was voluntary and was not linked to course completion, evaluation, or residency standing. The paper surveys were kept anonymous, without direct identifiers; responses were reported only in aggregate. In the survey, medical resident participants provided demographic information, including their level of training and specific residency program. Learners were asked to select responses of *yes* or *no* to the following items: this case presented during the simulation is relevant to my work; the simulation case was realistic; the

Copyright © 2026 Merritt et al. This is an open-access publication distributed under the terms of the Creative Commons Attribution license.

debrief promoted reflection and team discussion; the group discussion helped me develop and prioritize evaluation and management options for a child with bronchospasm; and the facilitators created a safe environment for discussion and exploration.

Confidence level was self-assessed before and after the simulation as a Kirkpatrick Level 2 learning outcome.[20] Confidence levels reflected participant ability to achieve the 3 primary educational objectives, with ratings based on a 3-point analytic rubric: 1 = *needs improvement*, 2 = *proficient*, 3 = *mastery* (Appendix G). Educational objectives were created based on Bloom's Taxonomy. While this rubric was not intended to function as a formal entrustability assessment, its structure and terminology were informed by concepts familiar to pediatric residents from competency-based medical education. The assessment scale was informed by the developmental framing used in pediatric residency training EPAs, with clear explanations for each level listed: "pre-entrustable" corresponds to a response of *needs improvement*, "entrustable" corresponds to a response of *proficient*, and "exceeds expectations" corresponds to a response of *mastery*. We used these simplified, more familiar labels to facilitate rapid self-assessment by participants. The scale was reviewed and refined through discussion with 2 pediatric attendings and 2 pediatric residents prior to implementation.

For all analysis, PGY-3 and PGY-4 residents were grouped together, due to small sample sizes. Only the pediatric residents completed the surveys and were included in the analysis. Descriptive statistics were used to summarize participant demographics. Paired *t* tests assessed changes in confidence scores. One-way analysis of variance (ANOVA) was used to evaluate differences in improvement by training level.

Results

Ninety-six residents participated in this simulation from April to May 2025 on 4 separate dates, with 90 residents included in the final analysis after excluding 6 participants for missing data. Seventy-two participants (80%) were categorical pediatric residents, 8 were pediatric neurology residents, 5 were triple board residents, and 5 were combined medicine-pediatric residents. The cohort included 32 PGY-1, 34 PGY-2, and 24 PGY-3 or PGY-4 residents.

All learners answered *yes* when asked if this case was relevant to their work and realistic. They all answered *yes* when asked whether the debrief promoted reflection and team discussion, whether the group discussion helped them develop and prioritize evaluation and management options, and whether the facilitators created a safe environment for discussion.

For identifying and treating bronchospasm (educational objective 1), resident learners' pre/postsimulation perceived confidence levels in attaining the objective (scores on a 3-point scale) were a mean (SD) of 2.0 (0.7) before the simulation and 2.7 (0.5) after the simulation. For escalating respiratory support (educational objective 2), resident mean confidence levels were 2.0 (0.7) before the simulation and 2.7 (0.4) after the simulation. For recognizing severe hypotension and identifying the correct vasopressor (educational objective 3), resident mean confidence levels were 1.9 (0.7) before the simulation and 2.7 (0.5) after the simulation. Confidence scores significantly improved across all 3 objectives ($P < .01$ for each; Table and Figure 1). At least one level of improvement in confidence in implementing educational objectives 1, 2, and 3 was seen in 62%, 67%, and 72% of residents, respectively, after participation in the simulation.

To evaluate differences in simulation-related improvement in confidence across training levels, a one-way ANOVA was conducted with PGY-1, PGY-2, and PGY-3/4 learners treated as independent groups. When the postsimulation improvement in mean confidence levels was examined across all objectives, there was a statistically significant difference by PGY level ($F = 5.06$, $P = .008$). Subgroup analysis by objective revealed that improvement in learners' perceived confidence in implementing educational objective 2 (escalation of respiratory support) varied significantly across PGY levels ($F = 6.01$, $P = .004$; Figure 2), whereas differences in confidence implementing educational objective 1 approached but did not reach statistical significance ($F = 2.93$, $P = .059$). No significant difference by PGY level was observed in learners' confidence in implementing educational objective 3 ($F = 1.29$, $P = .28$).

Discussion

Following a high-fidelity simulation focused on the management of near fatal bronchospasm, respiratory failure, and

Table. Participant ($N = 90$) Mean Confidence Scores in Implementing Each Educational Objective Before and After the Simulation of Near-Fatal Bronchospasm Complicated by Septic Shock in a Pediatric Patient

Objective[a]	Pre *M* (*SD*)	Post *M* (*SD*)	*P*
1: Identify and treat bronchospasm	2.03 (0.67)	2.67 (0.50)	<.0001
2: Escalate respiratory support	2.01 (0.73)	2.72 (0.45)	<.0001
3: Recognize severe hypotension and identify the correct vasopressor	1.94 (0.66)	2.68 (0.49)	<.0001

[a]Pediatric residents rated their confidence levels on a 3-point scale (1 = *needs improvement*, 2 = *proficient*, 3 = *mastery*).

Copyright © 2026 Merritt et al. This is an open-access publication distributed under the terms of the Creative Commons Attribution license.

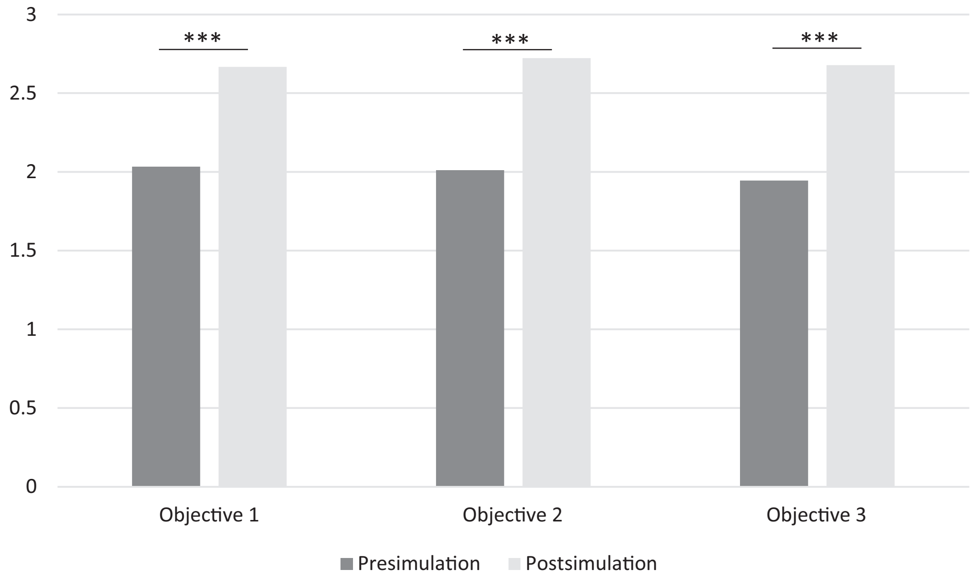

Figure 1. Participants' mean confidence scores before and after the simulation of near-fatal bronchospasm complicated by septic shock in a pediatric patient, based on perceived confidence in implementing the 3 educational objectives: (1) identify and treat bronchospasm, (2) escalate respiratory support, and (3) recognize severe hypotension and select the correct vasopressor (***$P < .0001$ for all comparisons, by paired *t* test). Pediatric residents rated their confidence levels on a 3-point scale: 1 = *needs improvement*, 2 = *proficient*, 3 = *mastery*.

uncompensated septic shock, pediatric residents reported increased confidence across all training levels in achieving our educational objectives. The most notable improvement was observed among PGY-1 residents, though improvements were observed across all levels, underscoring the simulation's broad educational value. These confidence gains reflect New World Kirkpatrick Level 2 learning,[20] in which changes in learners' attitudes, self-efficacy, and preparedness are recognized as

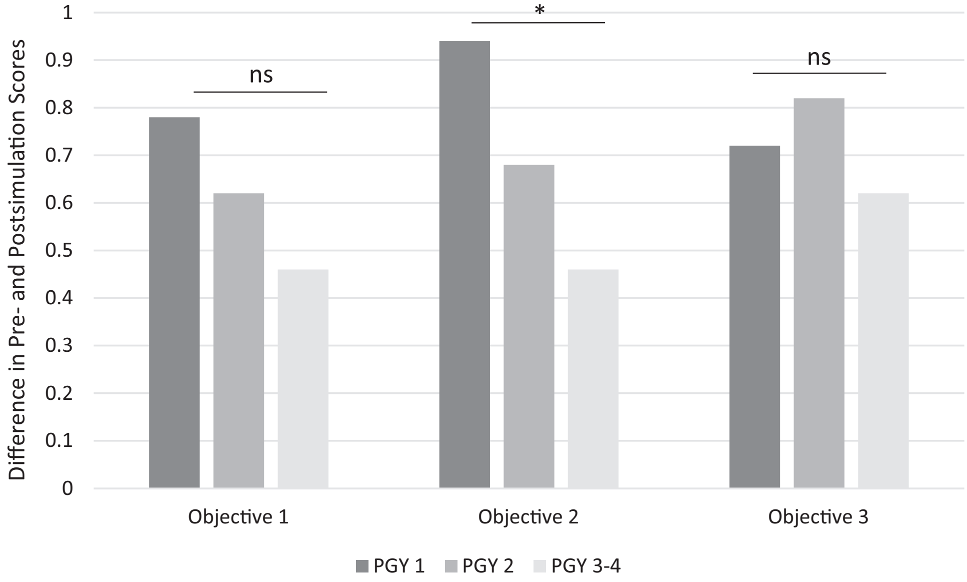

Figure 2. Mean improvement pre- to postsimulation in participants' confidence scores across PGY levels in implementing each educational objective: (1) identify and treat bronchospasm, (2) escalate respiratory support, and (3) recognize severe hypotension and select the correct vasopressor. Statistically significant differences in confidence scores by PGY level were observed only for implementing educational objective 2 (*$P < .01$). Objective 1 trended toward a significant difference between PGY levels ($P = .059$), while no significant difference by PGY level was observed for objective 3 ($P = .28$) (by one-way analysis of variance). Pediatric residents rated their confidence levels on a 3-point scale: 1 = *needs improvement*, 2 = *proficient*, 3 = *mastery.* Abbreviation: ns, not significant.

Copyright © 2026 Merritt et al. This is an open-access publication distributed under the terms of the Creative Commons Attribution license.

meaningful outcomes of educational interventions. Our findings highlight the value of simulation in bridging gaps in resident confidence for recognizing and treating rare but life-threatening pediatric emergencies.

The development and implementation of this case revealed important lessons for future educators. Case complexity required careful design to balance clinical realism with learner manageability. Refinements to case flow were made, such as pacing the evolution of clinical deterioration and staggering the introduction of diagnostic data, to ensure that learners could prioritize competing demands without becoming overwhelmed. Facilitated debriefing further allowed residents to consolidate clinical reasoning. These adjustments may be valuable for educators designing similarly multifaceted scenarios.

This scenario was conducted using a high-fidelity simulator, video monitor, and extensive medication formulary, which may limit generalizability to resource-restricted settings. However, this simulation can be adapted to a limited-resource setting by providing physical examination findings, vital signs, and laboratory and imaging results verbally, as well as by substituting verbal orders for medication administration and respiratory support escalation. The simulation was performed by pediatric medical residents but could be easily adapted to emergency medicine or pediatric intensive care trainees.

Additionally, the simulation evaluation relied on self-reported confidence levels as the primary outcome, which, while useful for capturing learner perception, do not objectively assess skill acquisition, preparedness, or long-term retention. Moreover, the confidence scale anchors may not have fully aligned with observed learner performance, as anticipated management errors included delayed recognition of evolving shock, whereas the lowest anchor on the scale assumed recognition of shock with limited intervention. Future implementations should consider refining the confidence scale levels (Appendix G) to better reflect the full spectrum of learner performance. Incorporating performance-based assessments using the checklist or repeated assessments over time could provide a more thorough assessment of learner competence and preparedness.

Future directions include adapting the simulation for longitudinal use, such as repeating it at the beginning and end of residency to assess knowledge retention and skill progression. Broader integration of this simulation into pediatric residency curriculum could strengthen preparedness for pediatric respiratory and septic emergencies, aligning with educational priorities to improve outcomes in high-risk, time-sensitive conditions.

Overall, our findings support the use of simulation as an effective tool to enhance pediatric resident knowledge, confidence, and preparedness. By targeting critical HALF clinical scenarios, it addresses an important educational gap and advances training consistent with Kirkpatrick Level 2 learning outcomes.

Appendices

A. Simulation Case with Critical Actions.docx
B. Simulation Environmental Preparation List.docx
C. Prebriefing Guide.docx
D. Data Slides.pptx
E. Debriefing Guide.docx
F. Postdebrief Handout.docx
G. Simulation Evaluation Form.docx

All appendices are peer reviewed as integral parts of the Original Publication.

Taylor Anne Merritt, MD: Fellow, Department of Pediatrics, Division of Hospital Medicine, The University of Texas Southwestern; ORCID: https://orcid.org/0009-0007-6950-7896

Annesha Dutta, MD: Resident, Department of Pediatrics, The University of Texas Southwestern

Yarah Ghotmi, MD: Fellow, Department of Pediatrics, Division of Palliative Care, The University of Texas Southwestern

Ngoc Van Horn, MD: Associate Professor, Department of Pediatric, Division of Emergency Medicine, The University of Texas Southwestern

Disclosures
None to report.

Funding/Support
None to report.

Prior Presentations
Merritt T, Van Horn N. A simulation-based approach to severe bronchospasm complicated by septic shock. Presented at: APPD Medical Education Conference; September 18, 2025; St. Louis, MO.

Ethical Approval
The University of Texas Southwestern Institutional Review Board reviewed this project.

References

1. Most recent national asthma data. Centers for Disease Control and Prevention. Updated May 2023. Accessed February 19,

Copyright © 2026 Merritt et al. This is an open-access publication distributed under the terms of the Creative Commons Attribution license.

2026. https://www.cdc.gov/asthma/most_recent_national_asthma_data.htm

2. Freedman MS, Forno E. Initial emergency department vital signs may predict PICU admission in pediatric patients presenting with asthma exacerbation. *J Asthma*. 2023;60(5):960-968. https://doi.org/10.1080/02770903.2022.2111686
3. Schlapbach LJ, Watson RS, Sorce LR, et al; Society of Critical Care Medicine Pediatric Sepsis Definition Task Force. International consensus criteria for pediatric sepsis and septic shock. *JAMA*. 2024;331(8):665-674. https://doi.org/10.1001/jama.2024.0179
4. Weiss SL, Fitzgerald JC, Balamuth F, et al. Delayed antimicrobial therapy increases mortality and organ dysfunction duration in pediatric sepsis. *Crit Care Med*. 2014;42(11):2409-2417. https://doi.org/10.1097/CCM.0000000000000509
5. Holzemer NF, Pomeranz ES, Tomlinson S. Improving the pediatric emergency department learning experience: a simulation-based orientation for pediatric PGY 1 residents. *MedEdPORTAL*. 2020; 16:10919. https://doi.org/10.15766/mep_2374-8265.10919
6. van Schaik SM, Von Kohorn I, O'Sullivan P. Pediatric resident confidence in resuscitation skills relates to mock code experience. *Clin Pediatr (Phila)*. 2008;47(8):777-783. https://doi.org/10.1177/0009922808316992
7. McGaghie WC, Issenberg SB, Cohen ER, Barsuk JH, Wayne DB. Does simulation-based medical education with deliberate practice yield better results than traditional clinical education? A meta-analytic comparative review of the evidence. *Acad Med*. 2011;86(6):706-711. https://doi.org/10.1097/ACM.0b013e318217e119
8. Saavedra HR, Turner JS, Cooper DD. Use of simulation to improve the comfort of pediatric residents managing critically ill emergency department patients. *Pediatr Emerg Care*. 2018; 34(9):633-635. https://doi.org/10.1097/PEC.0000000000001596
9. Cristallo T, Walters M, Scanlan J, Doten I, Demeter T, Colvin D. Multidisciplinary, in situ simulation improves experienced caregiver confidence with high-risk pediatric emergencies. *Pediatr Emerg Care*. 2021;37(9):451-455. https://doi.org/10.1097/PEC.0000000000001623
10. Cory MJ, Colman N, McCracken CE, Hebbar KB. Rapid cycle deliberate practice versus reflective debriefing for pediatric septic shock training. *Pediatr Crit Care Med*. 2019;20(5): 481-489. https://doi.org/10.1097/PCC.0000000000001891
11. Croft A, Barach P, Bona A, et al. A novel simulation-based Pediatric Respiratory Distress Educational Curriculum (PReDEC) for emergency medicine residents. *Pediatr Emerg Med J*. 2025;12(1):10-19. https://doi.org/10.22470/pemj.2024.01109
12. Adler M, Trainor J, Siddall V, McGaghie W. Pediatric acute care simulator case: status asthmaticus. *MedEdPORTAL*. 2009;5:1083. https://doi.org/10.15766/mep_2374-8265.1083
13. Adler M, Trainor J, Siddall V, McGaghie W. Pediatric acute care simulator case: shock (sepsis). *MedEdPORTAL*. 2008;4:821. https://doi.org/10.15766/mep_2374-8265.821
14. Reid J, Stone K. Pediatric emergency medicine simulation curriculum: status asthmaticus. *MedEdPORTAL*. 2014;10:9660. https://doi.org/10.15766/mep_2374-8265.9660
15. Reid J, Stone K. Pediatric emergency medicine simulation curriculum: septic shock. *MedEdPORTAL*. 2013;9:9639. https://doi.org/10.15766/mep_2374-8265.9639
16. Conlon L, Gillenwater E. Leukemia blast crisis: a simulation case for residents. *MedEdPORTAL*. 2016;12:10504. https://doi.org/10.15766/mep_2374-8265.10504
17. Roussin CJ, Weinstock P. SimZones: an organizational innovation for simulation programs and centers. *Acad Med*. 2017;92(8): 1114-1120. https://doi.org/10.1097/ACM.0000000000001746
18. Eppich W, Cheng A. Promoting Excellence and Reflective Learning in Simulation (PEARLS): development and rationale for a blended approach to health care simulation debriefing. *Simul Healthc*. 2015;10(2):106-115. https://doi.org/10.1097/SIH.0000000000000072
19. Post-Pre Survey Responses. Simon Fraser University Centre for Educational Excellence. Accessed February 19, 2026. https://www.sfu.ca/teaching-inquiry/inquiry-support/postpre.html
20. Kirkpatrick JD, Kirkpatrick WK. *Kirkpatrick's Four Levels of Training Evaluation: A New World*. Association for Talent Development; 2016.

Received: October 19, 2025
Accepted: February 9, 2026
Published: April 7, 2026

Copyright © 2026 Merritt et al. This is an open-access publication distributed under the terms of the Creative Commons Attribution license.
